# Construction of a novel molecular typing and scoring system for anoikis distinguishes between different prognostic risks and treatment responsiveness in low-grade glioma

**DOI:** 10.3389/fimmu.2023.1105210

**Published:** 2023-04-11

**Authors:** Ganghua Zhang, Aiyan Chen, Jianing Fang, Anshan Wu, Guanjun Chen, Panpan Tai, Haotian Chen, Xinyu Chen, Ke Cao

**Affiliations:** ^1^ Department of Oncology, Third Xiangya Hospital, Central South University, Changsha, China; ^2^ Zhuzhou Hospital Affiliated to Xiangya School of Medicine, Central South University, Zhuzhou, China

**Keywords:** low-grade glioma, anoikis, temozolomide, prognosis, immune cell infiltration, immunotherapy

## Abstract

**Background:**

The main factors responsible for low-grade glioma (LGG)s’ poor prognosis and treatment effectiveness include recurrence and malignant progression. A specific type of programmed cell death, known as anoikis, which is crucial for tumor invasion and metastasis, however, has not yet been investigated in LGGs.

**Methods:**

We downloaded data of 509 samples from the TCGA-LGG cohort, carried out cluster analysis for typing twice on the basis of 19 anoikis-associated genes, and the subtypes were evaluated the differences in clinicopathological and biological features. ESTIMATE and single-sample gene set enrichment analysis were employed to examine the immunological milieu of LGGs, and enrichment analysis was used to look into the underlying biological mechanisms in LGGs. Cox regression analysis and the Least Absolute Shrinkage and Selection Operator regression algorithm were used to create a prediction scoring system. The scoring system was used for classifying LGG into high- and low- anoikis riskscore (anoiS) groups. The impact of the anoiS on the prognosis, standard treatment, and immunotherapy of patients with LGG was assessed using survival analysis and drug sensitivity analysis. Cell experiments were employed for the verification of the differential expression between LGG cells and normal cells of the anoikis gene team that regard CCT5 as the core.

**Results:**

Based on the expression profiles of the 19 anoikis-associated genes, all individuals with LGG were classified into four subtypes and two macrosubtypes. The different macrosubtypes had significantly different biological characteristics, and the anoirgclusterBD subtype manifested a significantly bad prognosis and a high immune level of infiltration. And subsequent secondary genotyping also showed good prognostic discrimination. We further constructed an anoikis scoring system, anoiS. LGG patients having a high anoiS had a worse prognosis in comparison to those having a low anoiS. The high anoiS group exhibited larger levels of immune infiltration and superior immunotherapy efficacy than the low anoiS group. The high anoiS group was also more susceptible to temozolomide (TMZ) than the low anoiS group, according to a drug sensitivity analysis of TMZ.

**Conclusion:**

This study constructed a scoring system for predicting the prognosis of patients with LGG and their responsive to TMZ and immunotherapy.

## 1 Introduction

As stated by the World Health Organization’s 2016 classification based on histological type, diffuse gliomas are classified as low-grade gliomas (LGGs; grades II and III) and glioblastomas (GBMs; grade IV) (1, 2). Grade II LGGs are defined as astrocytomas and grade III as oligodendrogliomas. LGGs grow more slowly than high-grade gliomas. LGGs comprise approximately 7.6% of all primary brain tumors, and their median survival rate is between 4.7–9.8 years (3). Although the prognosis of LGGs is significantly better than that of GBMs, LGGs are highly susceptible to recurrence and metastasis (4), and 45–74% of these recurrent metastatic cases progress to GBMs in patients with LGGs (5). Unfortunately, the existing key marker gene status (including isocitrate dehydrogenase (IDH) mutations, 1p19q co-deletions, and O^6^-methylguanine-DNA methyltransferase (MGMT) promoter methylation) does not manifest clinically important significance for LGGs as for GBMs. Therefore, new molecular typing and scoring systems should be developed to more accurately differentiate between patients with different prognostic risks and therapeutic sensitivities to develop individualized and precise treatment plans for each patient with LGG.

Standard treatment for LGG, temozolomide (TMZ) or PCV regimens based chemotherapy, radiotherapy and surgery, has not been very effective in preventing tumor recurrence and progression (6). Yao et al. (2021) found that overall survival after surgery and radiotherapy had improved following chemotherapy in newly diagnosed high-risk IDH-mutant patients with LGG, respectively; however, a proportion of individuals treated with TMZ developed TMZ-induced hypermutation recurrent tumors (7). Radiotherapy can provide survival benefits for most patients with LGGs. However, the optimal timing of radiotherapy remains controversial. It is unclear whether radiotherapy should be used early in the postoperative period or delayed until tumor progression has occurred (8). The advent and refinement of immunotherapy has had a significant influence on cancer treatment. Many clinical studies on immunotherapeutic agents are currently underway to ascertain the safety and efficacy in the treatment of gliomas (9). The clinical trials comprised only GBM patients; however, GBM patients have not shown any survival benefits from nivolumab (NCT02550249) administration (10). The tumor immune microenvironment (TIME) has a key involvement in cancer progression and tumor immunity, as it harbors key factors that may alter the efficacy of immunotherapy. The function of the TIME in LGGs requires systematic investigation, which may bring novel options for improving survival benefits in radiotherapy-resistant patients (11). Studies have shown that IDH mutations are not only a disease-defining biomarker and oncogenic driver in glioma, but are also a neoantigen and modulator of glioma immune evasion and are associated with an immunosuppressive phenotype (12, 13). This shows that immunotherapy may have a positive impact on how patients with LGG are treated.

A particular type of planned apoptosis, referred to as anoikis, is brought on by a lack of intercellular adhesion and cell-extracellular matrix (ECM) adhesion, or by an erroneous form of adhesion, and it is connected to a number of necessary cellular functions, for instance, cell migration and invasion (14, 15). Anoikis is generally triggered by the interplay of two apoptotic pathways, which can happen when mitochondria are interfered with or cell surface death receptors are activated (16–18). Cancer cells can avoid anoikis and acquire resistance to anoikis, which allows them to survive and colonize distant sites. Anoikis is a key mechanism that takes part in cancer invasion and metastasis (19–21). In the tumorigenesis models of breast cancer, it has been found that the deletion of E-cadherin (also known as CDH1) encourages angiogenesis and anoikis resistance, which in turn contributes to the development of metastatic disease. Moreover, HGF promotes anoikis resistance in endometrial cancer cells by elevation of cyclooxygenase-2 (COX-2) expression that is dependent on the PI3K-Akt pathway. A number of cancers have also been shown to overexpress promyosin-related kinase B (TrkB, also known as NTRK2), a powerful and selective inhibitor of anoikis. TrkB transfection confers anoikis resistance by activating the PI3K-Akt pathway in a highly anoikis-sensitive rat intestinal epithelial cells. Furthermore, in mammary epithelial cells, CDH1 acts synergistically with EGFR and ERBB2 protects cells from anoikis (22). CCT5 markedly promotes gastric cancer anti-anoikis to promote gastric cancer lymph node metastasis formation (23). In addition, several previously studies reported RAN, KIF11, ECT2, GDH1, and PLAG1 were related to anoikis resistance (24–27). And six datasets (GSE145806, GSE106592, GSE155457, GSE40690, GSE55958, GSE39220, and GSE40171) identified six anoikis-related genes. We then selected 19 ANOIRGs from those previously published articles (14, 22–27) and those six datasets (GSE145806, GSE106592, GSE155457, GSE40690, GSE55958, GSE39220, and GSE40171). Recent studies have shown that 27 anoikis-associated genes, based on gene set enrichment analysis (GSEA) screening of GBM, can predict patient prognosis and response to immunotherapy (28). Anoikis genes contribute to carcinogenesis, tumor invasion, and tumor infiltration despite the fact that few researchers have thoroughly evaluated their significance in LGGs. Our hypothesis was that LGGs develop a malignant phenotype and become anoikis resistant, which may explain their poor prognosis and aggressive metastatic spread.

First, we looked at 19 anoikis-related genes’ (ANOIRGs) differential expression and prognostic significance in LGGs. Then, based on 19 ANOIRGs, we developed new molecular typing using the Cancer Genome Atlas (TCGA) and China Glioma Genome Atlas (CGGA) databases, and we used ESTIMATE and single-sample gene set enrichment analysis (ssGSEA) algorithms to examine the intra-tumoral immune infiltrative landscape of LGGs. For predicting patient prognosis and responsiveness to TMZ treatment, an anoikis risk score (anoiS) was devised based on the anoikis potentially related genes (APRGs) identified from the screen. Studying anoikis-related gene expression patterns contributes to the personalization and improvement of treatment strategies for LGG patients by deepening our understanding of the aggressiveness of LGG.

## 2 Materials and methods

### 2.1 Data collection and processing

The GDC database’s (https://portal.gdc.cancer.gov/) TCGA-LGG cohort, which contains 509 LGG samples, was downloaded. Clinical information, FPKM values for gene expression, and RNA sequencing information were received from GDC. For further investigation, the FPKM values were subsequently transformed to transcripts per kilobase million (TPM) values (29). **Table 1** displays the TCGA-LGG cohort’s starting data. The mRNA expression profiles of normal brain tissue were acquired from the Genotype-Tissue Expression Project (GTEx, https://www.gtexportal.org). Data of a total of 527 simple nucleotide variation from the GDC database were downloaded. Tumor mutation burden (TMB) (mut/mb) = total number of mutations (including synonymous and nonsynonymous point mutations, substitutions, insertions, and deletions mutations)/size of the target region coding area. For each sample, TMB values were computed taking into consideration the definition of TMB. From UCSC Xena (https://xenabrowser.net/datapages/), data for 533 gene copy number variants (CNVs) were retrieved. The CGGA database (http://www.cgga.org.cn) was used to download the gene expression profiles and clinical information for the CGGA cohort. The IMvigor210 dataset was obtained in order to assess the anoikis riskscore’s ability to forecast the immunotherapy response. Using previously released studies (14, 22–27) and datasets(GSE145806, GSE106592, GSE155457, GSE40690, GSE55958, GSE39220, and GSE40171), we chose 19 ANOIRGs.

**Table 1:** Baseline Data Sheet of the TCGA-LGG cohort.

Characteristic	Levels	N (%)
Age	>45 years old	204 (39.6%)
	≤45 years old	311 (60.4%)
Gender	Male	285 (55.3%)
	Female	230 (44.7%)
Grade	G2	249 (48.4%)
	G3	265 (51.6%)
histological_type	Oligodendroglioma	191 (37.1%)
	Oligoastrocytoma	130 (25.2%)
	Astrocytoma	194 (37.7%)

### 2.2 Multi-omics analysis based on 19 ANOIRGs

Mutation annotation format (MAF) files of TCGA mutation data were subjected to analysis utilizing the “maftools” R package, and waterfall plots were drawn to visualize the mutations of the 19 ANOIRGs in the TCGA-LGG cohort. A CNV landscape of 19 ANOIRGs was developed based on CNV data from the TCGA-LGG cohort. To examine the differences in mRNA expression between normal and LGG samples, a differential analysis of the 19 ANOIRGs based on the LGG integrated expression profiles of GTEx and TCGA was carried out. The samples were separated into low and high expression groups by employing an optimal cutoff value of the gene expression profile, and comparison of the difference in overall survival (OS) between the low and high expression groups was made using the log-rank test and univariate Cox regression. A co-expression prognostic network of 19 ANOIRGs was built employing univariate cox regression analysis and Pearson’s correlation analysis.

### 2.3 Unsupervised clustering based on 19 ANOIRGs

Unsupervised consistency clustering and classification based on 19 ANOIRGs was attempted *via* the “ConsensusClusterPlus” R package (30), re-sampling 80% of the samples 50 times using coalescent pam clustering with Euclidean distance. After that, the differences in OS between various subtypes were compared by employing the Kaplan-Meier (K-M) survival analysis, the expression of the 19 ANOIRGs between subtypes was compared using box line plots, and comparison of the distribution of the 19 ANOIRGs’ expression across subtypes was made using the t-Distributed Stochastic Neighbor Embedding (t-SNE). In an attempt to display the distribution of the 19 ANOIRG expressions and clinicopathological characteristics across the various subtypes, heat maps were produced using the “pheatmap” R package.

### 2.4 Gene set variation analysis

Molecular Signature Database (MsigDB, http://software.broadinstitute.org/gsea/msigdb/) was employed for obtaining data for the Kyoto Encyclopedia of Genes and Genomes (KEGG) pathway, HALLMARK pathway and Reactome pathway and “c2.cp.kegg.v7.5.1. symbols.gmt”, “h.all.v7.5.1.symbols.gmt” and “c2.cp.reactome.v7.5.1.symbols.gmt” was obtained as the reference gene set. We subsequently performed Gene Set Variation Analysis (GSVA) (31) on various subtypes using the “GSVA” R package to examine the variation in the biological processes of different subtypes and visualize it using a heatmap.

### 2.5 Infiltration estimation of the immune microenvironment

The StromalScore, ImmuneScore, and ESTIMATEScore were computed using the “ESTIMATE” R program. The quantitative metrics known as the ImmuneScore and the StromalScore, which measure the quantity of stromal and immune components, respectively, are obtained from gene expression profiling data. Also, the two scores are added to create the ESTIMATEScore, which has a negative correlation with tumor purity (32). Using the ssGSEA method based on the “GSVA” R package, the enrichment score of 23 immune cells in the TIME was then calculated, which is a depiction of the relative infiltration abundance of individual immune cells (33).

### 2.6 Screening for differentially expressed genes and enrichment analysis

Using |logFoldChange|>1 and FDR<0.05, differentially expressed genes (DEG) were screened between various subtypes. Gene Ontology (GO) and KEGG functional enrichment analyses were performed by employing the “clusterProfiler” R package (34), and statistically significant results can be represented by adjusted p-value <0.05.

### 2.7 Second unsupervised clustering based on differentially expressed prognostic genes with strong prognostic significance

DEGs with p <0.05 were screened using univariate Cox regression analysis, and genes with differentially expressed prognostic genes with strong prognostic significance (SDEPGs) were subsequently selected based on a threshold of |1-HR| >0.5. Forest plots of the results from the Cox regression analysis, were drawn using the “forestplot” R package. A secondary unsupervised cluster analysis was then performed based on SDEPGs, classifying the samples into different subtypes with identical specific clustering parameters. Subsequently, we compared the OS differences between subtypes using survival analysis, compared the differential expression of 19 ANOIRGs between subtypes using box-line plots, and plotted heatmaps to show the distribution of SDEPGs expression and clinicopathological features between the subtypes.

### 2.8 Construction and validation of anoiS

The “glmnet” R package was used to execute the Least Absolute Shrinkage and Selection Operator (LASSO) regression analysis, which reduces the dimensionality of high-dimensional data by capping the sum of the absolute values of coefficients at less than a set threshold. Only genes with non-zero coefficients in the LASSO regression analysis were chosen for additional investigation since the coefficients of the relatively tiny contributing variables were zero. We increased the stability and reproducibility of the LASSO model by adding a random seed. Then, the “randomForest” R package was used to screen genes for anoikis characteristics. The default iteration number of random forest algorithm is 100. When 500 trees are constructed, the model is considered to be robust enough. Based on Gini coefficient method, the “important” function was used to score for genes screened by LASSO model, and genes with a score above two were proceeded for further analysis. Finally, the genes obtained were screened using the multivariate Cox proportional risk regression analysis to obtain 12 potentially relevant anoikis genes that were identified as the best predictive traits and named as APRGs. These genes were selected to further calculate the anoiS for each patient using a multivariate Cox regression model: anoiS = ho(t) * exp (β 1X1+ β _2_X2+…. + β nXn). In the equation mentioned, β denotes the regression coefficient, and ho(t) refers to the baseline risk function. Multivariate Cox regression model was constructed from the “predict” function of the “rms” R package. Using the median anoiS, patients from the TCGA database were split into high- and low-anoiS groups. To ascertain the clinical independence of the anoiS for prognostic prediction, univariate and multivariate Cox analyses were utilized. The K-M survival analysis was then used to assess the differences in OS and progression-free survival (PFS) between LGG patients with high and low anoiS. Receiver operating characteristic (ROC) analysis and area under the curve (AUC) values were utilized for evaluating the prediction accuracy of the anoiS for 1-year, 3-year, and 5-year OS and PFS. The calibrate function of the “rms” R package plots the calibration curve, with a maximum resampling sample size of 1000. PFS and OS calibration curves for 1, 3, and 5 years were drawn. The forecast output of the model matches the actual one more closely the closer the calibration curve is near the line “y=x”. Last but not least, Sankey plots were created with the “ggalluvial” R package to show the relationship between various subtypes, an anoiS group, and prognosis. Comparison of the variations in anoiS between subtypes was achieved *via* box plots.

### 2.9 Exploration of anoiS-based immune microenvironment and immunotherapy

Through the correlation matrix, the ssGSEA algorithm was employed for quantifying the infiltration abundance of 23 different kinds of immune cells in the TIME, to show the correlation among the anoiS and immune cell infiltration levels, as well as investigate any potential relationships between the APRGs and the anoiS and 46 immune checkpoints. Finally, the prognostic predictive effect of the anoiS in the immunotherapy population was investigated using a survival analysis based on the IMvigor210 cohort.

### 2.10 AnoiS-based mutational analysis

First, we created distinct mutation landscapes for the high and low anoiS groups by making use of the “Maftools” R package. We analyzed variance to examine the variations in TMB levels between the high and low anoiS groups based on the TMB values of each TCGA-LGG cohort sample and Spearman’s correlation analysis to investigate the relation between anoiS and TMB. The optimal cutoff value was then determined using the “surv cutpoint” and “surv categorize” functions of the “survminer” R package and was taken into consideration as the boundary. Survival analysis was then conducted for the comparison of the differences in OS between patients with different anoiS and TMB statuses. The sample was then divided into high and low TMB groups.

### 2.11 Clinical subgroup analysis based on anoiS

“Age,” “gender,” “grade,” and “histological type” were selected as the clinical subgroup characteristics of patients with LGG, the distribution ratio of different clinical subgroup characteristics were counted in the high and low anoiS groups, and explored the differences of the anoiS among patients with different clinical subgroup characteristics.

### 2.12 Validation and exploration of the CGGA cohort

First, the prognostic differences between the whole population, chemotherapy alone, radiotherapy alone, and radiochemotherapy population were compared using the K-M survival analysis based on OS data. Next, the potential association of anoiS with three classical genetic statuses, 1p19q co-deletion, IDH mutation, and MGMT promoter methylation, was determined using differential analysis and correlation analysis, and the AUC values of ROC curves were employed for comparing the predictive efficacy of the three gene statuses, anoiS, and grade. For 1-, 3-, and 5-year OS survival, the corresponding calibration curves were plotted. Finally, we compared the differences of OS among people with different treatment modalities and genetic status in the high and low anoiS groups, respectively.

### 2.13 Temozolomide sensitivity analysis

The sensitivity to TMZ in patients with LGG was predicted using the “pRRophetic” R package (35) and the “oncopredict” R package (36) by predicting the IC50 value of temozolomide, ridge regression model was constructed to predict the AUC value of TCGA cohort by the “oncopredict” R package based on the expression profile data and AUC data of GDSC2 database. the lower the value of IC50 or AUC, the greater the sensitivity of the patient to TMZ. Differences in drug sensitivity to TMZ among the low and high anoiS groups were subjected to comparison, and correlations between the IC50 values of the anoiS and TMZ were demonstrated using Spearman’s correlation analysis. Correlation matrices were constructed to visualize the correlation between the 19 ANOIRGs with 12 APRGs and the IC50 values of TMZ.

### 2.14 Screening of key genes for anoikis and potential reciprocal genes

Based on the correlation matrix of genes with predicted IC50 values of TMZ, the genes with the largest negative correlation coefficients, when correlated with the IC50 values of TMZ obtained from the combination of the two algorithms, were screened from the 19 ANOIRGs. The results of the combined differential analysis and prognostic analysis were excluded for obtaining the anoikis key genes (AKGs), and the gene co-expression network was constructed by employing AKG and the 12 APRGs with a correlation coefficient |r|>0.5 as the threshold to screen APRGs closely related to AKG as their potential reciprocal genes by using Pearson’s correlation coefficient. Additionally, the Human Protein Atlas (HPA; https://www.proteinatlas.org/) database was used to acquire the protein level immunohistochemical (IHC) staining results for chaperonin containing TCP1 subunit 5 (*CCT5*) between normal and LGG tissues.

### 2.15 Cell culture

The Cell Bank of Type Culture Collection of the Chinese Academy of Science (Shanghai, China), supplied the human Hs683 low-grade glioma cell line employed in the current work. American Type Culture Collection (ATCC, Manassas, VA, USA) provided human astrocytes (NHA) and human SW1088 low-grade glioma cell lines. Hs683 cells were cultured in Dulbecco’s modified Eagle’s medium (DMEM; HyClone, Logan, USA) supplemented with 10% fetal bovine serum (FBS; Gibco, NY, USA) and 1% penicillin-streptomycin (HyClone, Logan, USA) at 37°C with 5% CO_2_. 10% Fetal bovine serum (FBS; Gibco, NY, USA) was supplemented to Leibovitz’s (L)-15 media to develop a culture media for SW1088 cells. The fetal bovine serum (FBS; Gibco, NY, USA), 15%, and 1% penicillin-streptomycin (HyClone, Logan, USA) were added as supplements to the Dulbecco’s modified Eagle’s medium (DMEM; HyClone, Logan, USA) for the NHAs’ culture.

### 2.16 Quantitative reverse transcription polymerase chain reaction

A faster reagent (Invitrogen) was used to extract total RNA from cultured cells. PrimeScript RT Reagent Kit (TaKaRa, Shiga, Japan) was employed to create cDNA from isolated RNA, and SYBR Green PCR Master Mix was utilized for quantitative reverse transcription polymerase chain reaction (qRT-PCR). GAPDH was employed as an internal loading control, and expression levels were quantified by employing the 2^–ΔΔCt^ method. GraphPad Prism version 9.0.1 (GraphPad Software, San Diego, California USA, www.graphpad.com) was used for the visualization of qRT-PCR results and a two-sample unpaired t-test. Tsingke Biotech (Tsingke, China) synthesized the complete set of primers used for qRT–PCR. **Supplementary Table 2** lists all the primer sequences used in this work.

### 2.17 Statistical analysis

R version 4.2.1 (R Foundation for Statistical Computing, Vienna, Austria) was used to perform analyses, and the Perl language, which was mainly used for batch cleaning and collation of the data. The “limma” R package (37) was employed for DEGs screening. The Wilcoxon test was applied for differential analysis to compare the two groups in the bioinformatics analysis part. For the Analysis of Variance, the Kruskal-Wallis test was employed for comparisons involving more than two groups. The Spearman’s correlation coefficient was employed in this study’s correlation analysis unless otherwise noted. The survival of the various patient groups was compared using the K-M survival analysis and the log-rank test. A two-tailed p value < 0.05 was regarded as statistically significant for all statistical analyses.

## 3 Results

### 3.1 Mutational landscape, transcriptional alteration, and prognostic value of ANOIRGs in LGGs

**Figure 1:**
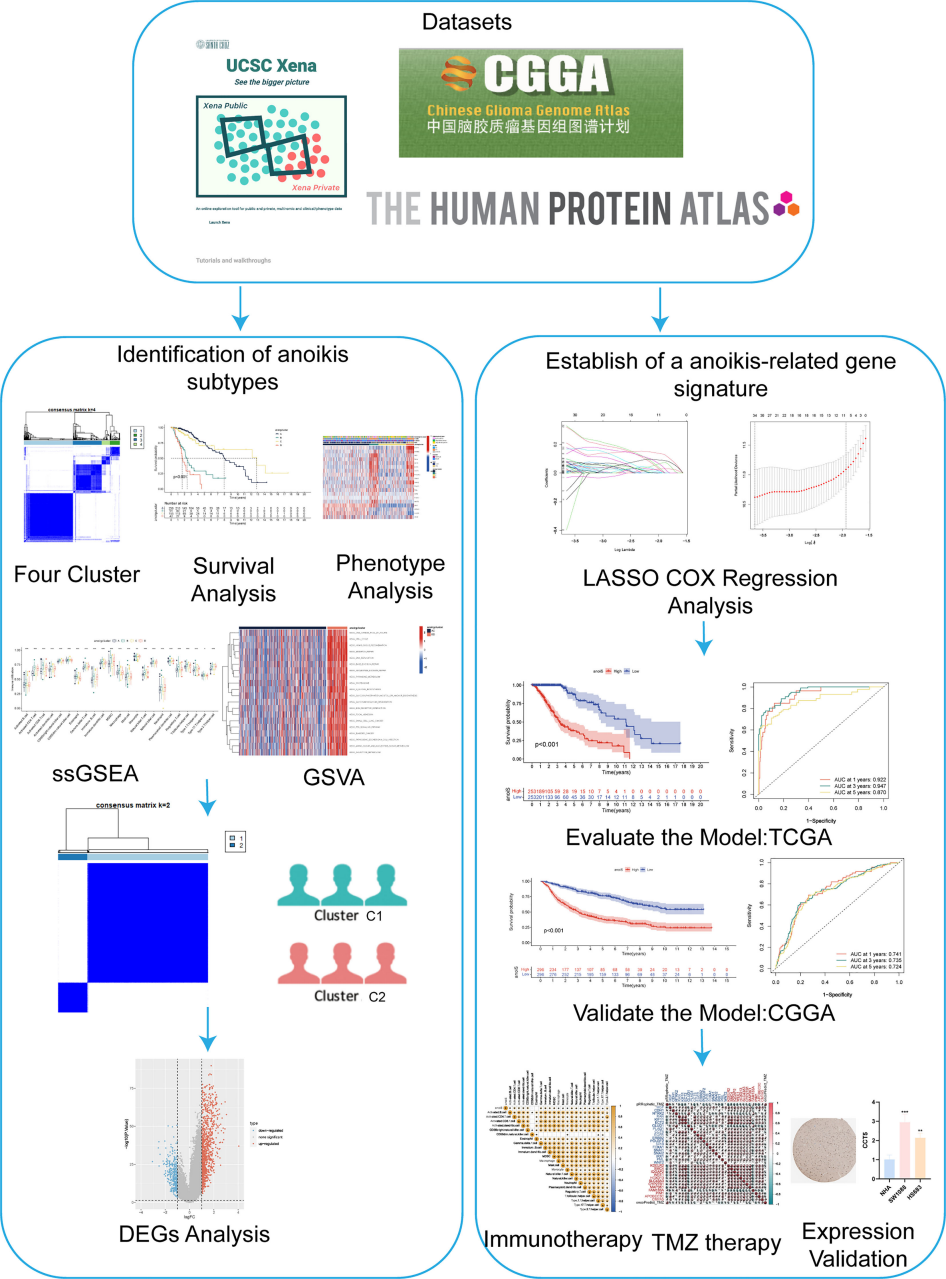
The flowchat of this study.

**Figure 1** illustrates the workflow of this study. The differential expression and prognostic significance of 19 ANOIRGs in LGGs were analyzed using the combined expression profiles of the TCGA and GTEx cohorts and survival prognostic data of the TCGA cohort. The findings demonstrated that *PLAG1* and *SNAI1* were expressed at low levels in LGGs compared to normal tissues, and the remaining 17 ANOIRGs were highly expressed in LGGs (**Supplementary Figure 1A**). The co-expression prognostic network of the 19 ANOIRGs showed that *GLUD1* and *NTRK2* were protective factors based on Univariate Cox regression analysis, whereas the remaining 17 ANOIRGs were LGG risk factors. Broadly speaking, 17 risk ANOIRGs positively correlated with each other, whereas two protective ANOIRGs correlated negatively with each other (**Supplementary Figure 1B**). The K-M survival analysis showed that the remaining 18 ANOIRGs had a significant effect on the prognosis of patients with LGG except for WNT2 (p < 0.05; **Supplementary Figure 1C**). Mutation analysis showed that ANOIRGs-related mutations occurred in 43 of 523 samples, with an incidence of 8.22% (**Supplementary Figure 2A**). Among the 43 mutations, *EGFR* had the highest mutation frequency of 6%, mainly missense mutations (**Supplementary Figure 2A**). The outcome of CNV analysis showed that *SPIB* had the highest copy number deletion frequency, while *EGFR* had the highest increase in copy number frequency (**Supplementary Figure 2B**). Finally, we constructed a CNV landscape of 19 ANOIRGs at chromosomal loci (**Supplementary Figure 2C**).

### 3.2 Identification of anoikis related subtype in LGGs

**Figure 2:**
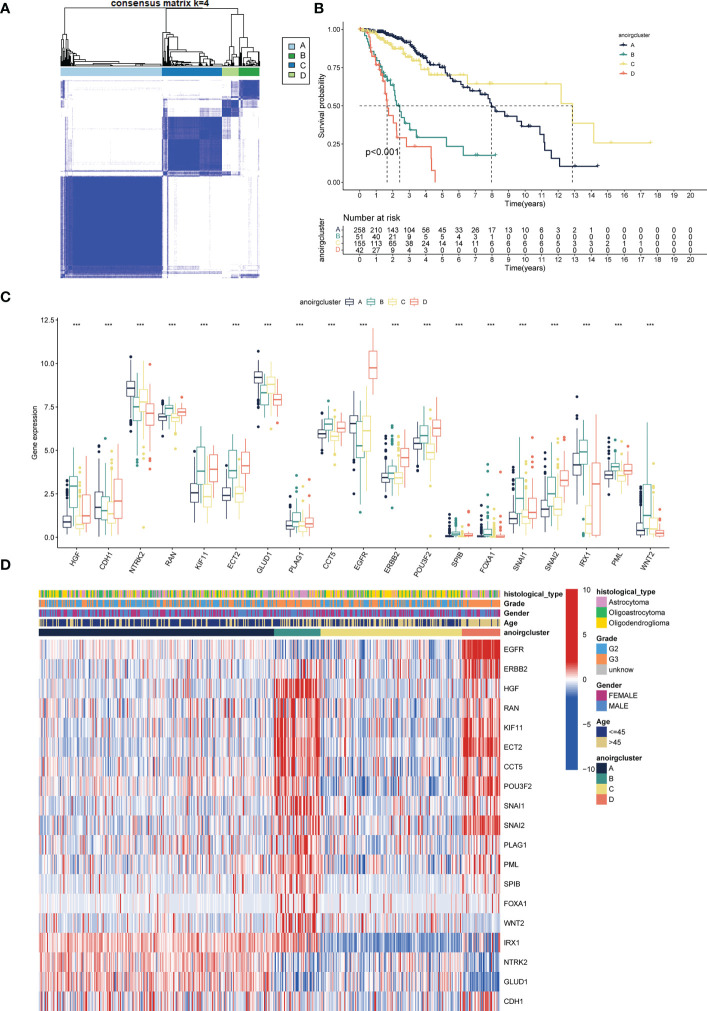
Identification of anoikis related subtypes and exploration of the clinical and biological features of subtypes. **(A)** Unsupervised consensus clustering divides LGG samples into four clusters (k=4) based on 19 ANOIRGs. **(B)** OS curves for the four subtypes of patients with LGG. **(C)** Expression differences analysis of 19 ANOIRGs among the four subtypes. **(D)** Difference distribution of clinicopathological features and ANOIRGs expression among the four subtypes. ANOIRGs, anoikis-related genes; ***p < 0.001.

TCGA-LGG cohort samples were clustered according to the consistent expression of the 19 ANOIRGs into four different modification patterns representing four different molecular subtypes (**Figure 2A**). The consensus clustering results are shown in **Supplementary Figures 3A–C**. The K-M survival analysis revealed significant differences in OS among the four anoikis related subtypes, with patients in anoirgclusterA and anoirgclusterC having a better prognosis than those in anoirgclusterB and anoirgclusterD (p < 0.001, **Figure 2B**). In addition, we plotted box plots (**Figure 2C**) and heatmaps (**Figure 2D**) to visualize the differential expression of 19 ANOIRGs in different molecular subtypes and found that the expression of *HGF*, *KIF11*, *ECT2*, *CCT5*, *ERBB2*, *POU3F2*, *SPIB* and *SNAI2* was higher in anoirgclusterB and anoirgclusterD than in anoirgclusterA and anoirgclusterC (p < 0.01). Compared with the histological type, there were mostly astrocytomas in anoirgclusterB and anoirgclusterD and oligoastrocytomas and oligodendeogliomas in anoirgclusterA and anoirgclusterC. Grade 3 was also the most common grade in anoirgcluster B and anoirgcluster D, while grade 2 was mostly observed in anoirgcluster A and anoirgcluster C (**Figure 2D**). Furthermore, GSVA was employed to compare the enriched pathway of both macrosubtypes of anoirgclusterA and anoirgclusterC, and anoirgclusterB and anoirgclusterD in the KEGG pathway (**Supplementary Figure 4A**), HALLMARK pathway (**Supplementary Figure 4B**), and Reactome pathway (**Supplementary Figure 4C**), and detected significant pathway variations between them (primarily enriched in cell cycle-related pathways).

### 3.3 Characterization of TIME in different anoikis related subtypes

Patients with different anoikis related subtypes exhibited t-SNE-based ANOIRGs’ expression distinguishability feature (**Supplementary Figure 5A**). Then, we applied stromal, immune, and ESTIMATE scores to all LGG samples to gauge the degree of TIME cell infiltration in various subtypes. According to **Supplementary Figure 5B**, anoirgclusterB and anoirgclusterD outperformed anoirgclusterA and anoirgclusterC in terms of StromalScore, ImmuneScore, and ESTIMATEScore (p < 0.001). Additionally, we employed ssGSEA for quantifying the infiltrating abundance of 23 immune cells and investigate the distinct patterns of the immune-infiltrating landscape of the four subtypes in order to characterize the immune cell infiltration in various anoikis-related subtypes. anoirgclusterB and anoirgclusterD manifested a higher infiltration level of activated B, dendritic, CD4 T, CD8 T, CD56 bright natural killer, macrophages, gamma delta T, immature B, mast, dendritic, natural killer, natural killer T, regulatory T, plasmacytoid dendritic, type 1 T helper, type 2 T helper, T follicular helper, and myeloid-derived suppressor cells (MDSCs) than anoirgclusterA and anoirgclusterC (p <0.05, **Supplementary Figure 5C**). The outcome suggests that anoirgclusterB and anoirgclusterD have an elevated degree of stromal and immune cell infiltration than anoirgclusterA and anoirgclusterC. Based on these results, we found that anoirgclusterAC and anoirgclusterBD have similar ANOIRG expression profiles and prognostic and immune infiltration characteristics, which supports our classification of samples into two macrosubtypes: anoirgclusterAC and anoirgclusterBD.

### 3.4 Identification and secondary clustering of DEGs among macrosubtypes

**Figure 3:**
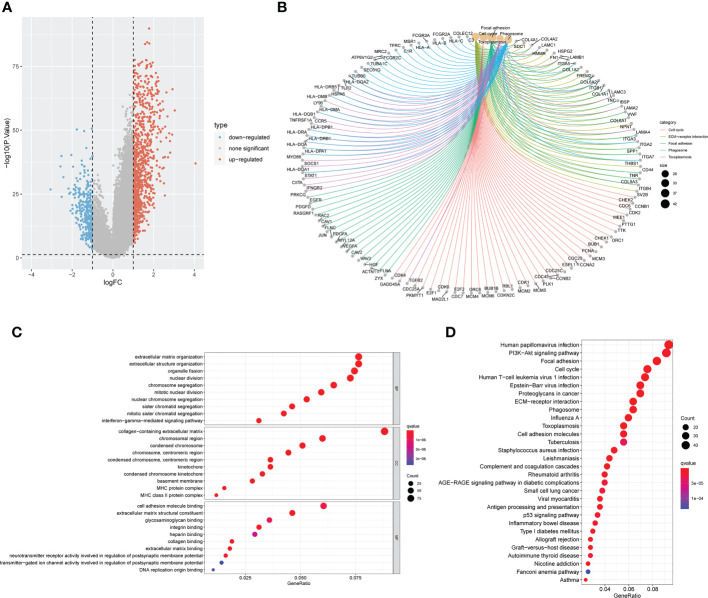
Screening and enrichment analysis of DEGs between the two anoikis related macrosubtypes. **(A)** Volcano plot of differential analysis between the two macrosubtypes to identifity DEGs. **(B)** The network diagram shows the corresponding relationship between the five KEGG pathways with the lowest p-value and related genes. **(C)** KEGG enrichment analysis of DEGs between the two macrosubtypes. **(D)** GO enrichment analysis of DEGs between the two macrosubtypes. DEGs, differentially expressed genes; ANOIRGs, anoikis-related genes.

For an additional investigation on the potential biological behavior of various anoikis subtypes, we carried out a differential analysis of the two anoikis macrosubtypes. DEGs with |logFoldChange| >1 and p < 0.05 were then screened, and 1251 DEGs were identified. Volcano plots showed that DEGs mainly expressed in the large anoirgclusterBD subtypes were highly expressed (p < 0.05, **Figure 3A**). KEGG (**Figure 3C**) and GO (**Figure 3D**) enrichment analysis was conducted for these DEGs, showing that the top five pathways were based on adjusted p-values in KEGG and the corresponding network of relationships with associated genes (p < 0.05, **Figure 3B**). A number of pathways are associated with the cell cycle.

**Figure 4:**
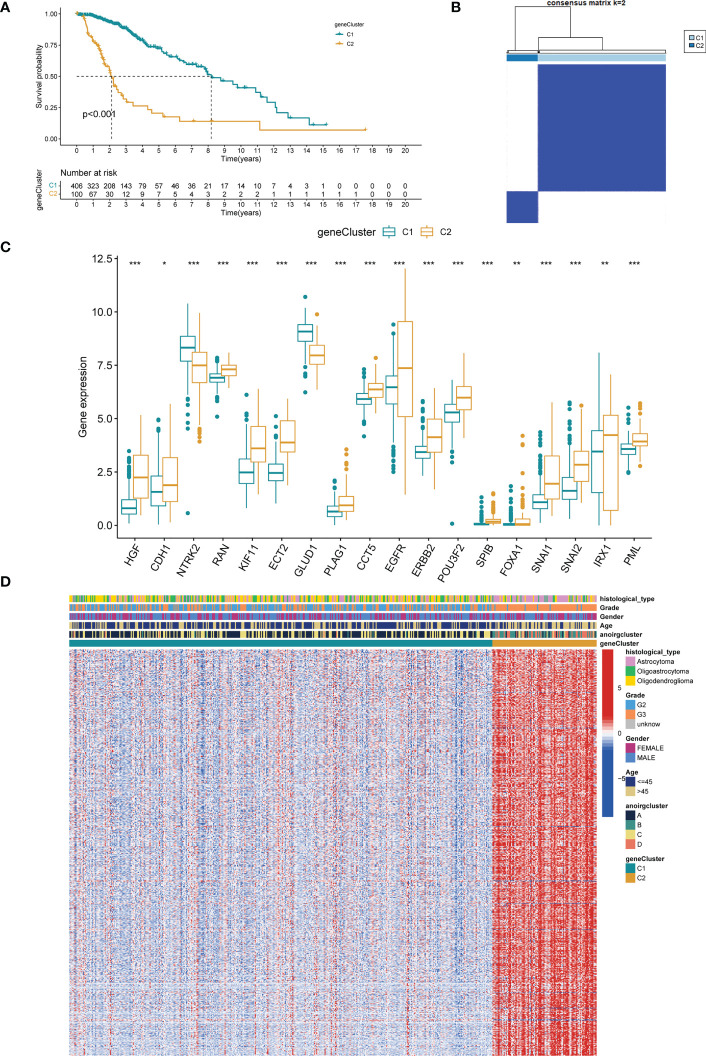
Identification of two gene-subtypes and exploration of the clinical and biological features of gene-subtypes. **(A)** Unsupervised consensus clustering divides LGG samples into two gene-clusters base on 669 SDEPGs (k=2). **(B)** OS curves for the two gene-subtypes of patients with LGG. **(C)** Expression differences analysis of 18 significative ANOIRGs between the two gene-subtypes. **(D)** Difference distribution of clinicopathological features and ANOIRGs expression between the two gene-subtypes. ANOIRGs, anoikis-related genes; SDEPGs, differentially expressed prognostic genes with strong prognostic significance; *p < 0.05; **p < 0.01; and ***p < 0.001.

A univariate Cox regression analysis on 1251 DEGs associated with anoikis was performed for the identification of DEGs with prognostic significance for LGG, yielding 1218 differentially expressed prognostic genes. A further 669 genes (SDEPGs) were selected based on the criterion of |1-HR| >0.5. Based on the 669 SDEPGs, secondary clustering was performed two clusters were identified: genecluster C1 and C2 (**Figure 4B**). **Supplementary Figures 3D–F** presents the clustering results. As depicted by the K-M survival curves, a significantly better prognosis was predicted for genecluster C1 in comparison to genecluster C2 (p < 0.001, **Figure 4A**). Eighteen ANOIRGs had significantly different expressions between genecluster C1 and C2 (p < 0.001, **Figure 4C**). In addition, a heatmap was employed to show the distribution of expression of 669 SDEPGs and clinicopathological characteristics for both geneclusters (**Figure 4D**).

### 3.5 Construction of the anoiS in LGGs

**Figure 5:**
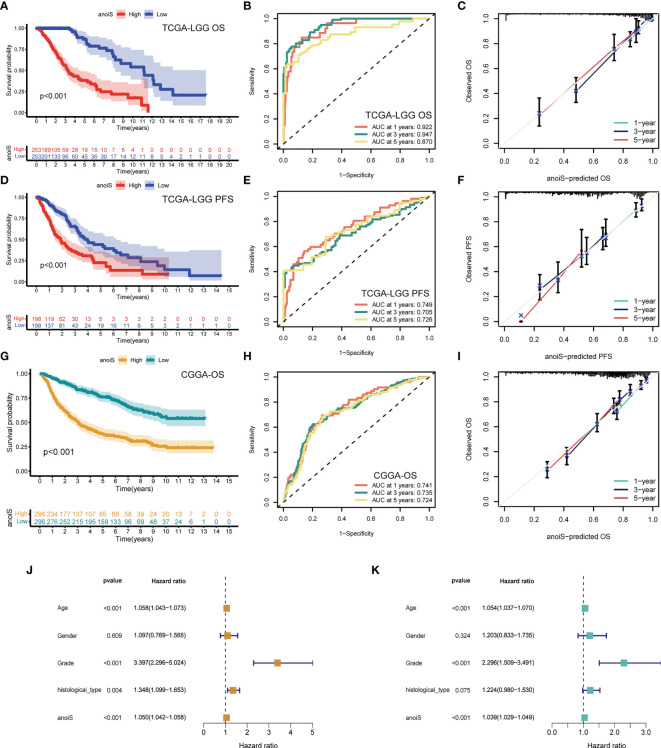
Construction and validation of anoiS. **(A)** OS analysis between the high- and low-anoiS groups in the TCGA-LGG cohort. **(B)** ROC curves of anoiS in predicting OS at 1-, 3-, and 5-years in the TCGA-LGG cohort. **(C)** Calibration curve to verify the predictive value of anoiS regarding the 1-, 3-, and 5-year OS in the TCGA-LGG cohort. **(D)** PFS analysis between the high- and low-anoiS groups in the TCGA-LGG cohort. **(E)** ROC curves of anoiS in predicting PFS at 1-, 3-, and 5-years in the TCGA-LGG cohort. **(F)** Calibration curve to verify the predictive value of anoiS regarding the 1-, 3-, and 5-year PFS in the TCGA-LGG cohort. **(G)** OS analysis between the high- and low-anoiS groups in the CGGA cohort. **(H)** ROC curves of anoiS in predicting OS at 1-, 3-, and 5-years in the CGGA cohort. **(I)** Calibration curve to verify the predictive value of anoiS regarding the 1-, 3-, and 5-year OS in the CGGA cohort. **(J, K)** Verification of the clinical independence of anoiS by univariate Cox regression analysis **(J)** and multivariate Cox regression analysis **(K)**. anoiS, anoikis riskscore.

First, we performed LASSO regression analysis for downscaling screening based on 669 SDEPGs (**Supplementary Figures 6A, B**) and obtained a total of 34 genes. We then calculated the gene importance scores based on the Gini coefficient method in random forest, selected genes with scores ≥2 as disease signature genes (**Supplementary Figures 6C, D**), obtained 20 genes to enter the multivariate cox regression analysis, and finally screened 12 APRGs to construct the anoikis scoring system anoiS. The results of the multivariate Cox regression analysis for the 12 APRGs are shown in **Supplementary Table 1**. Among them, *KDELR2*, *SMC4*, *IQGAP2*, *WEE1*, *HOXD13*, *SLC43A3*, *CYP27B1*, *MAP3K1*, *PIM1*, and *APOBEC3C* were highly expressed in LGGs (**Supplementary Figure 7A**), and their high expression was associated with poor prognosis (**Supplementary Figure 7B**). Next, we divided the cohort samples into high- and low-anioS groups based on the median anioS. The K-M survival analysis showed that patients in the high anoiS group had poorer OS and PFS than those in the low anoiS group (p < 0.001, **Figures 5A, D**). The ROC curves also showed strong predictive accuracy of anioS, with AUCs for OS at 1, 3, and 5 years of 0.922, 0.947, and 0.870 for OS, respectively (**Figure 5B**), and AUCs of 0.749, 0.705 and 0.726 for PFS at 1, 3 and 5 years, respectively (**Figure 5E**). It was discovered that the anoiS has good accuracy in predicting OS and PFS at 1, 3, and 5 years in patients with LGG by using the calibration curve to test the predictive utility of the model (**Figures 5C, F, I**). We also used the CGGA dataset to confirm the reliability and stability of the 12-gene signature prediction model. The prognosis of the high anoiS group was considerably poorer than that of the low anoiS group, according to a survival analysis (p < 0.001, **Figure 5G**). With AUCs of 0.741, 0.735, and 0.724 at 1, 3, and 5 years, respectively, the ROC curve data showed that anoiS had a significant prognostic prediction potential (**Figure 5H**). The anoiS appeared to be a clinically independent risk prognostic factor for patients with LGG in the TCGA-LGG cohorts, according to univariate and multivariate Cox regression analyses paired with other clinical subgroup features (p < 0.001, **Figures 5J, K**). Sankey plots revealed a correlation between the prognosis, anioS, and subtype (**Supplementary Figure 6E**). AnoirgclusterB and anoirgclusterD had a higher chance of matching geneclusterC2, higher anioS, and a worse prognosis in LGG patients. Boxplots were also used to confirm this conclusion. (p < 2.22e-16, **Supplementary Figures 6F, G**).

### 3.6 Clinical subgroup analysis based on the anoiS

**Figure 6:**
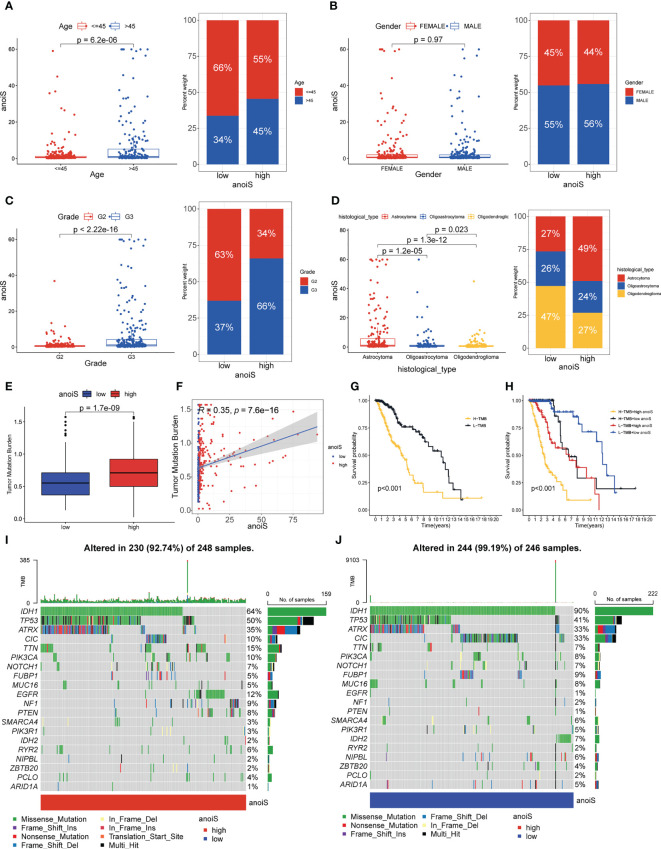
Clinical subgroup and tumor mutation analysis base on anoiS in the TCGA-LGG cohort. **(A–D)** Relationship exploration between clinical features and anoiS by difference comparison and ratio distribution: age **(A)**, gender **(B)**, grade **(C)** and histological type **(D, E)** Differential analysis of TMB between the high- and low- anoiS groups. **(F)** Spearman correlation analysis between anoiS and TMB. **(G)** OS analysis of patients with high- and low- TMB. **(H)** OS analysis among four groups of patients stratified by the anoiS and TMB. **(I, J)** Tumor mutation landscape in the high- and low- anoiS groups. anoiS, anoikis riskscore.

Stacked histograms were employed to demonstrate the percentage of individual clinical characteristics in the high and low anoiS groups and box plots were plotted to show the variations in the anoiS between various clinical subgroup features, in order to further investigate the relationship between the anoiS and clinical subgroup characteristics. Patients older than 45 years demonstrated a higher risk in comparison to those younger than 45 years (p = 6.1e-06, **Figure 6A**), the anoiS did not show significant differences between gender (**Figure 6B**), anoiS was higher in patients graded G3 than in those with G2 (p < 2.22e-16, **Figure 6C**), and patients with astrocytomas had higher anoiS than those with oligoastrocytomas and oligodendeogliomas (p < 0.05, **Figure 6D**).

### 3.7 Mutation correlation analysis of high and low anoiS populations

We further investigated TMB’s connection to the anoiS because it is intimately tied to tumor immunity. The findings revealed a positive correlation between TMB and anoiS (**Figure 6F**) and that TMB in the high anoiS group was higher in comparison to that in the low anoiS group (**Figure 6E**). Additionally, the samples were split into high and low TMB groups using the optimal cutoff value as the boundary, and it was discovered that the high TMB group’s prognosis was poorer than the low TMB group’s (**Figure 6G**). Patients with high TMB and anoiS demonstrated the poorest prognosis, whereas those with low TMB and anoiS had the best prognosis, according to a combined study of the effect of TMB and anoiS status on patient prognosis (**Figure 6H**).

To elucidate the potential genomic mutational mechanisms associated with anoikis, we used waterfall plots to depict the mutational landscape in the high and low anoiS populations; *IDH1*, *TP53* and *ATRX* had a higher mutation frequency in the cohort, with mutation rates of 64%, 50%, and 35% in the high anoiS group (**Figure 6I**) and 90%, 41%, and 33% in the low-anoiS group, respectively (**Figure 6J**). The mutation rates of *IDH1* and *IDH2* were lower in the high anoiS group than in the low anoiS group, while the mutation rate of *TP53* was higher than that in the low anoiS group.

### 3.8 Exploring the association between anoiS and immune infiltration landscape

**Figure 7:**
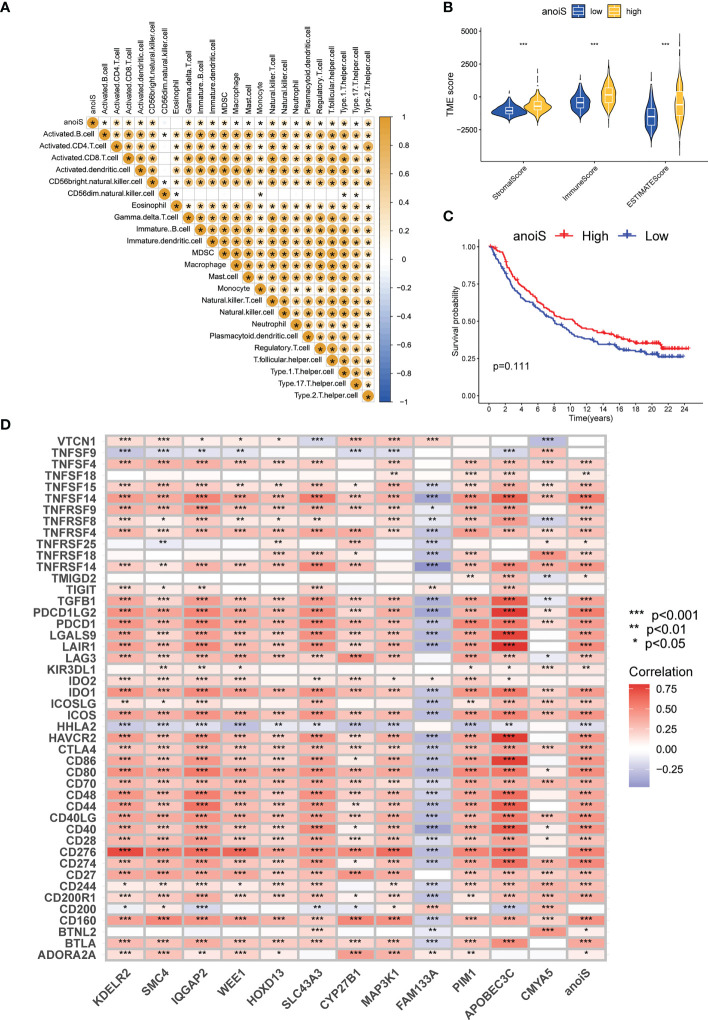
Immune microenvironment and immunotherapy efficacy analysis based on anoiS. **(A)** The spearman correlation matrix between anoiS and infiltration level of 23 immune cells. Yellow means positive correlation, whereas blue means negative correlation. **(B)** Violin plot for difference comparison of StromalScore, ImmuneScore, and ESTIMATEScore between the low- and high-anoiS groups. **(C)** OS analysis based on anoiS grouping in the IMvigor210 Cohort. **(D)** The spearman correlation matrix among APRGs, anoiS and 46 ICGs. Red means positive correlation, whereas blue means negative correlation. APRGs, anoikis-potential related genes; anoiS, anoikis riskscore; ICGs, immune checkpoint genes; *p < 0.05; **p < 0.01; and ***p < 0.001.

A correlation matrix (**Figure 7A**) was constructed for investigating the association between anoiS and immune cell infiltration, and we found that there was a substantial positive correlation between the two (p < 0.05, with the exception of CD56 dim natural killer cells and Eosinophils). In **Supplementary Figure 8A**, findings for particular individual cells have been displayed. With the utilization of ESTIMATE to assess the infiltration characteristics of the tumor microenvironment in LGG patients, it was found that the StromalScore, ImmuneScore, and ESTIMATEScore were substantially higher in the high anoiS group than in the low anoiS group (p < 0.05, **Figure 7B**). Thereafter, the association between anoiS and immunotherapy efficacy was verified using the IMvigor210 immunotherapy cohort. The outcome implies that individuals with a high anoiS had a good prognosis (**Figure 7C**). We additionally evaluated the correlation between the level of expression of 12 APRGs and the infiltration level of 23 immune cells. As shown in **Supplementary Figure 8B**, among the 12 APRGs, *FAM133A* negatively correlated with most immune cells (except CD56 dim natural killer cells and Eosinophils), *CMYA5* negatively correlated with Eosinophils, whereas positively correlated with activated CD56 dim natural killers, CD4+T cells, CD8+T cells, type 1 T helper cells, and type 2 T helper cells, plasmacytoid dendritic cells. *CYP27B1* negatively correlated with CD56 dim natural killer cells, type 1 T helper cells, Eosinophils, and Monocytes, whereas positively correlated with CD56 bright natural killers, activated CD4+T cells, and type 2 T helper cells. The remaining nine APRGs positively correlated with most immune cells (except CD56 dim natural killer cells, type 1 T helper cells, Eosinophils, and Monocytes). Next, we evaluated the correlation between the 12 APRGs and 46 immune checkpoint genes (ICGs). Most of the 12 APRGs positively correlated with a number of immune checkpoints, such as *PD-1*, *PD-L1*, *CTLA-4*, *TIM-3*, *B7-H3*, *IDO1*, and *LAG3*, while *FAM133A* negatively correlated with *CTLA-4*, *TIM -3*, *PD-1*, *PD-L1*, *B7-H3*, *IDO1*, and *LAG3*, and anoiS positively associated with most immune checkpoints, such as *CTLA-4*, *TIM-3*, *PD-1*, *PD-L1*, *B7-H3*, *IDO1*, and *LAG3* (**Figure 7D**).

### 3.9 Exploring the association between treatment modality and genetic status

**Figure 8:**
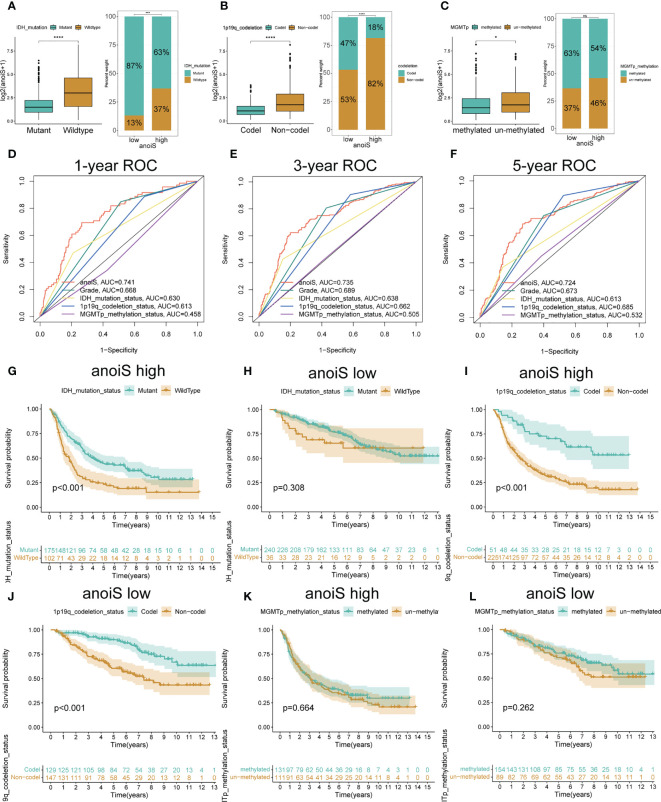
Validation and exploration of anoiS in the CGGA cohot. **(A-C)** Relationship exploration between three genetic status and anoiS by difference comparison and ratio distribution: IDH mutation status **(A)**, 1p19q codeletion status **(B)**, and MGMT methylation status **(C)**. **(D-F)** ROC curves of anoiS and other LGG prognostic factors (grade, IDH mutation status, 1p19q codeletion status, and MGMTp methylation status) in predicting OS at 1- **(D)**, 3- **(E)**, and 5-years **(F)** in the CGGA cohort. **(G-L)** OS analysis showing the effects of IDH mutation status **(G, H)**, 1p19q codeletion status **(I, J)** MGMT methylation status **(K, L)** on prognosis of the high- and low- anoiS groups. *p < 0.05, ****p < 0.0001.

The baseline data for the CGGA-LGG cohort is presented in **Table 2**. The proportion of individual gene status in the high and low anoiS groups was shown using stacked histograms and boxplots were plotted to show the difference in the anoiS between gene statuses. The findings depict that individuals with the IDH mutation (p<0.001, **Figure 8A**) and 1p19q co-deletion (p<0.001, **Figure 8B**) had lower anoiS, and patients placed in the high anoiS group manifested a lower proportion of IDH mutations (p<0.001, Figure 8A) and 1p19q co-deletion (p<0.001, **Figure 8B**). Patients with MGMT non-methylation had higher anoiS than MGMT-methylated patients, while MGMT methylation status did not vary substantially between patients in the low and high anoiS groups (p=0.024, **Figure 8C**). The comparison of clinical characteristics showed that the anoiS was the highest and MGMT promoter methylation was the lowest in AUC values at 1, 3, and 5 years of OS (**Figures 8D–F**)

**Table 2:** Baseline Data Sheet of the CGGA-LGG cohort.

Characteristic	Levels	N (%)
Age	>45 years old	160 (27.0%)
	≤45 years old	432 (73.0%)
Gender	Male	341 (57.6%)
	Female	251 (42.4%)
Grade	WHO II	270 (45.6%)
	WHO III	322 (54.4%)
histological_type	A	160 (27.0%)
	O	106 (17.9%)
	OA	9 (1.5%)
	AA	206 (34.8%)
	AO	91 (15.4%)
	AOA	20 (3.4%)
IDH_mutation	Wildtype	138 (25.0%)
	Mutant	415 (75.0%)
1p19q_codeletion	Non-codel	372 (67.4%)
	Codel	180 (32.6%)
MGMTp_methylation	Un-methylated	200 (41.2%)
	Methylated	285 (58.8%)

A, astrocytoma; O, oligodendroglioma; OA, oligoastrocytoma; AA, anaplastic astrocytoma; AO, anaplastic oligodendroglioma; AOA, anaplastic oligoastroytoma.

In the high anoiS group, the prognosis of patients in the IDH mutant group was better in comparison to that of patients in the IDH wildtype group, as shown by the survival analysis (p < 0.001, **Figure 8G**); in the low anoiS group, IDH mutant group and the patients placed in the IDH wildtype group, did not exhibit a significant difference in terms of the prognosis (Figure 8H). For 1p19q co-deletion, survival analysis demonstrated that the prognosis of patients in the 1p19q co-deletion group was improved in comparison to that of patients in the 1p19q co-deletion negative group, regardless of high or low anoiS (**Figures 8I, J**). For MGMT methylation status, regardless of the high and low anoiS, no significant difference existed in terms of patients’ prognosis in the MGMT methylation group and MGMT non-methylation group as depicted by the survival analysis (**Figures 8K, L**).

**Figure 9:**
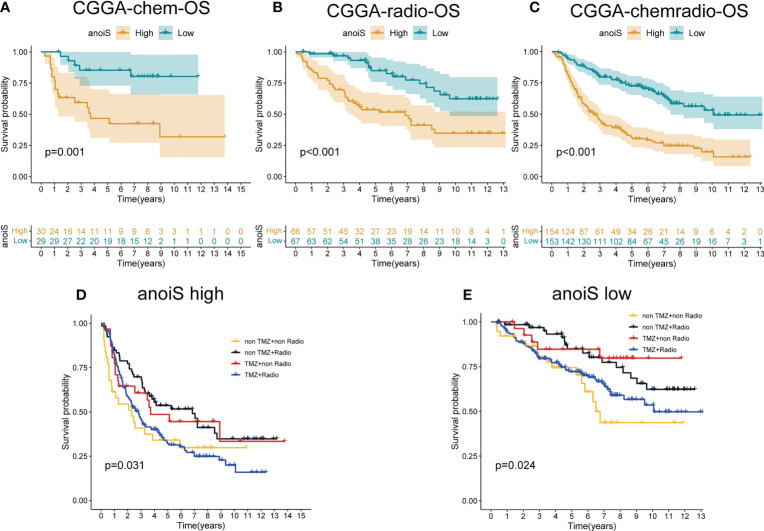
The potential association exploration of anoiS with Standard therapy sin LGGs. **(A-C)** OS analysis of the high- and low- anoiS groups in the CGGA chemotherapy cohort **(A)**, the CGGA radiotherapy cohort **(B)**, and the CGGA radiochemotherapy cohort **(C)**. **(D, E)** OS analysis among four patient groups stratified by the TMZ and radiotherapy in the high- anoiS group **(D)** and low- anoiS group **(E)**.

In addition, the prognosis was predicted separately for the different treatment modalities using the anoikis scoring system, and survival analysis depicted that patients placed within the high anoiS group manifested a significantly worse prognosis in comparison to the ones in the low anoiS group in the chemotherapy alone (p = 0.001, **Figure 9A**), radiotherapy alone (p < 0.001, **Figure 9B**), and radiochemotherapy (p < 0.001, **Figure 9C**) groups. Additionally, we found that for patients treated with radiotherapy alone or chemotherapy alone, the prognosis of tended to be better than that of the no-treatment and radiotherapy populations in both the high anoiS group (p = 0.031, **Figure 9D**) and the low anoiS group (p = 0.024, **Figure 9E**).

### 3.10 Prediction of TMZ sensitivity and screening of key gene teams

**Figure 10:**
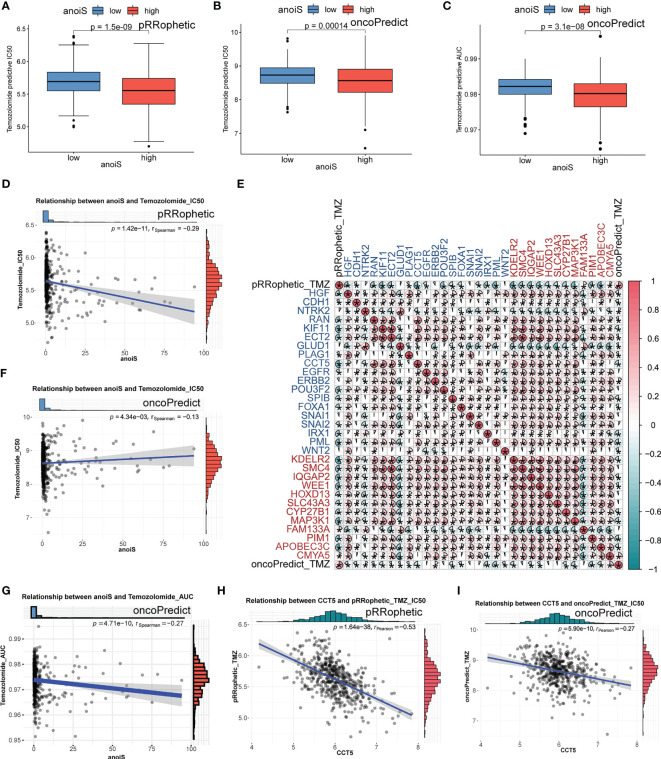
TMZ sensitivity analysis in LGGs of anoiS, ANOIRGs and APRGs. **(A, B)** Comparison of predictive IC50 value of TMZ between the high- and low- anoiS group by “pRRophetic” R package **(A)** and “oncopredict” R package **(B)**. **(C)** Comparison of predictive AUC value of TMZ between the high- and low- anoiS group by “ oncopredict “ R package. **(D, F)** Correlation analysis between anoiS and predictive IC50 value of TMZ by “pRRophetic” R package **(D)** and “oncopredict” R package **(F)**. **(E)** The pearson correlation matrix to show the relationship among predictive IC50 value of TMZ, 19 ANOIRGs and 12 APRGs. Red means positive correlation, whereas blue means negative correlation. **(G)** Correlation analysis between anoiS and predictive AUC value of TMZ by “oncopredict” R package. **(H, I)** Pearson correlation analysis between the expression of CCT5 and predictive IC50 value of TMZ by “pRRophetic” R package **(H)** and “oncopredict” R package **(I)**. ANOIRGs, anoikis-related genes; APRGs, anoikis-potential related genes. *p < 0.05.

**Figure 11:**
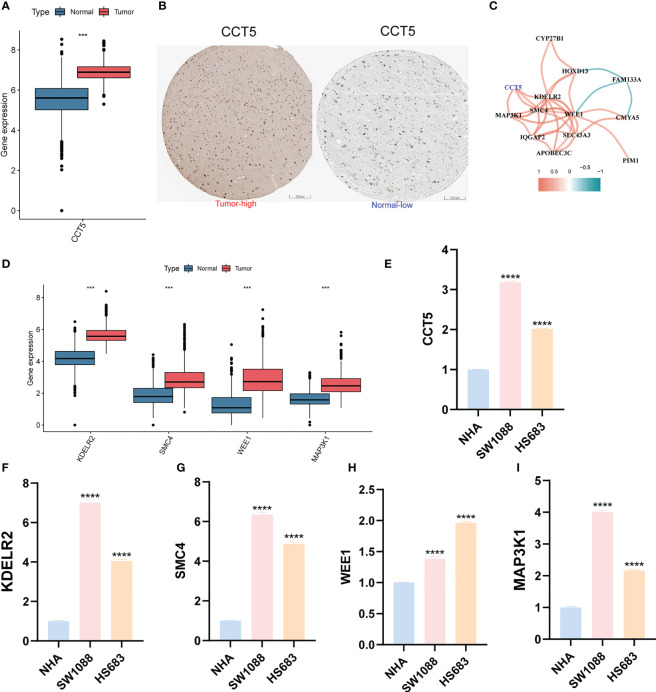
Differential expression validation of CCT5 and 4 most relevant APRGs between LGG and normal samples. **(A)** The expression differential analysis of CCT5 between LGG and normal brain tissues based on TCGA-Gtex integrated expression profile. **(B)** The HPA database showed the expression of CCT5 at the tissue protein level by immunohistochemistry. Scale bars correspond to 200μm. **(C)** Correlation network between CCT5 and all 12 APRGs. The cutoff value of the correlation coefficient for generating a line between genes was 0.5. **(D)** The expression differential analysis of KDELR2, SMC4, MAP3K1 and WEE1 between LGG and normal brain tissues based on TCGA-Gtex integrated expression profile. **(E-I)** The mRNA expression differential analysis of CCT5 **(E)**, KDELR2 **(F)**, SMC4 **(G)**, WEE1 **(H)**, and MAP3K1 **(I)** in LGG cells (SW1088, Hs683) and human astrocytes (NHA) by qRT-PCR. ANOIRGs, anoikis-related genes; APRGs, anoikis-potential related genes; ***p < 0.001, ****p < 0.0001. qRT-PCR data are means ± SD, with n = 3. Error bars represent the SD of triplicate experiments.

We first predicted the IC50 values of TMZ in LGG samples using both “pRRophetic” and “oncoPredict” algorithms to reflect the sensitivity of patients to TMZ. Patients in the high anoiS group demonstrated lower IC50 values of TMZ (**Figures 10A, B**) and lower AUC values of TMZ (**Figure 10C**), The IC50 values (**Figures 10D, F**) and AUC values (**Figure 10G**) of TMZ were negatively correlated with the anoiS. We then constructed a correlation matrix of 19 ANOIRGs and 12 APRGs using TMZ IC50 values. CCT5 showed the strongest negative correlation with TMZ IC50 values among the 19 ANOIRGs (**Figure 10E**) and was a differential prognostic gene (**Figures 11A, B**), which plays a potential cancer-promoting role in LGGs. Therefore, we regarded *CCT5* as the key gene for the study. We selected *CCT5* and 12 APRGs for constructing a gene co-expression network and found that *CCT5* had a strong positive correlation with *KDELR2*, *WEE1*, *SMC4* and *MAP3K1* (r > 0.5; **Figure 11C**). These four anoikis potential genes also showed differential prognosis, exerted potential pro-cancer effects (**Figure 11D**), and negatively correlated with the TMZ IC50 values. Therefore, we considered *CCT5* and these four anoikis genes as potential gene teams. The key gene *CCT5* showed the strongest negative correlation with the IC50 value of TMZ (**Figures 10H, I**). Additionally, qRT-PCR was used to detect the expression differences of *CCT5* (**Figure 11E**), *KDELR2* (**Figure 11F**), *SMC4* (**Figure 11G**), *WEE1* (**Figure 11H**), and *MAP3K1* (**Figure 11I**) between NHA and LGGs cells (SW1088 and Hs683). In LGGs cells, the expression of these genes demonstrated a notable up-regulation in comparison to that in human astrocytes (NHA) (p <0.05).

## 4 Discussion

It is well known that LGGs have a better clinical prognosis than GBMs. However, after standard treatment, most patients with LGG develop recurrence and metastasis, and eventually even GBMs (38). There is still a lack of effective molecular typing and therapeutic targets to help clinicians differentiate between LGG patients with different prognostic risks and treatment responsiveness. Anoikis, a specific type of apoptotic death caused by cell loss or inappropriate cell adhesion, has a close association with tumor invasion and metastasis. Nevertheless, tumor cells evade anoikis through multiple factors that regulate anoikis resistance, leading to uncontrolled growth of these cancer cells at other sites (20). Resistance to anoikis has been reported to be associated with the ability of GBMs to invade, metastasize, and develop drug resistance (39). Nevertheless, only a few works have focussed on the precise function of anoikis-associated genes in predicting LGG prognosis and their effects on LGG aggressive metastatic ability and TMZ drug resistance.

We selected 19 ANOIRGs as a starting point for our study. Most of these were anti-anoikis genes, which are highly expressed in LGGs and are risk prognostic factors. It is suggested that anti-anoikis can indeed result in poor patient prognosis amongst individuals suffering from LGG. Based on the expression profiles of the 19 ANOIRGs, all patients with LGG were classified into four subtypes and two macrosubtypes. The different macrosubtypes had significantly different biological characteristics, and the anoirgclusterBD subtype had a significantly bad prognosis and a high immune level of infiltration. After differential and prognostic analyses, we found a large number of SDEPGs, and subsequent secondary genotyping also showed good prognostic discrimination. Ultimately, using machine learning and screening of prognostic patterns, we constructed a robust and high-power anoikis scoring system, anoiS. It serves as an independent prediction system to accurately distinguish between patients with different survival and recurrence risks. The AUC value for OS prediction in the TCGA cohort was approximately 0.9, which was better than the existing common predictors. In contrast, a feature consisting of five LGG relapse and progression-associated genes had an AUC value of around 0.8 for OS prediction in the TCGA cohort (40), and another feature consisting of six TMB-associated genes also had an AUC value of around 0.8 for OS prediction in the TCGA cohort (41), which was lower than the AUC value in our study. The high predictive power of the risk score model is evident from the ROC, and the results of the calibration curve also suggest that the risk score model’s predictions corroborate quite well with the actual.

The extent of immune cell infiltration in patients with LGG in the high anoiS group had a significantly higher proportion than in the low anoiS group, and the level of immune cell infiltration manifested a positive correlation with the anoiS, according to our subsequent assessment of the immune microenvironment infiltration landscape. Patients having high anoiS levels tended to be more sensitive to immunotherapy in the immunotherapy cohort IMvigor210. TMB has been demonstrated to be successful in a variety of tumor types, such as lung cancer, and is frequently regarded as a novel biomarker enabling response prediction to cancer immunotherapy (42). According to Wang et al. (2019), patients with a greater TMB may have a better prognosis if they receive immunotherapy for several malignancies (43).. In contrast, our study’s findings showed that LGG patients with a high TMB had a poor prognosis. The superimposed effect of TMB and anoiS gave the worst prognosis to those with a high TMB and anoiS, but this group might have better immunotherapy efficacy. This also suggests that the poor efficacy of immunotherapy in LGGs may be due to the existence of other potential pathways that cause an irreversible poor prognosis in patients with high levels of immune infiltration.

Genetic tests for the 1p19q co-deletion, IDH mutation, and MGMT methylation are required for GBMs because they help predict a patient’s prognosis and treatment sensitivity. However, in LGGs, these three genes did not show superior efficacy. Yet, it has been demonstrated that LGG patients who also had a 1p/19q co-deletion and an IDH mutation had the best clinical results (44). Patients with 1p/19q co-deletion in LGGs demonstrated a longer OS and better treatment response in comparison to patients with 1p/19q intact (45, 46). Studies have suggested the use of MGMT status assessment as an adjunct to assess prognosis (47), but there are no clear guidelines or recommendations.

In the CGGA cohort, we explored the association between different genetic statuses and treatment modalities with the anoiS. The National Comprehensive Cancer Network (NCCN) guidelines (48) state, TMZ and radiotherapy as the most important treatment modalities for patients with LGGs. The anoiS significantly predicted the prognostic risk across treatment modalities in all populations. By combining the three gene statuses for further analysis, it was found that patients with low anoiS demonstrated an improved prognosis, possibly associated with an increased probability of IDH mutations and 1p19q co-deletions. Similarly, the mutational landscape of the TCGA cohort demonstrated that individuals in the low anoiS group exhibited a higher proportion of IDH mutations. The ROC curves suggest that the predictive efficacy of our anoiS exceeds that of grade and the three gene statuses, and is expected to be a new first-line predictor of LGGs. After patients are classified into high and low anoiS groups using the anoiS, other indicators can be analyzed to further differentiate the prognostic risk. Continued testing for IDH mutations is recommended for individuals placed within the high anoiS group, while testing for this is not recommended for patients placed within the low anoiS group. The testing of 1p19q is required for patients in both high and low anoiS groups. In contrast, the detection of MGMT promoter methylation has no significant prognostic prediction for either high or low anoiS in patients.

The sensitivity of patients to TMZ was predicted by determining the TMZ IC50 value and AUC value in LGG samples based on the two algorithms. We found that patients in the high anoiS group demonstrated a higher TMZ sensitivity, which positively correlated with the anoiS. In addition, almost all ANOIRGs and APRGs showed a positive correlation with TMZ sensitivity. We identified a core team of key genes, which included *CCT5*, which exhibited synergistic pro-oncogenic and TMZ-sensitizing effects in LGGs. CCT5, together with other homologous subunits (TCP1, CCT2, CCT3, CCT4, CCT6A, CCT6B, CCT7, and CCT8), forms a large molecular weight complex, chaperonin containing TCP-1 (TCP (T-complex protein 1) ring complex (TRiC), CCT/TRiC) (49). *CCT5* markedly promotes gastric cancer cell proliferation, anoikis, invasion, and lymphatic tube formation (23). CCT5 interacts with cell cycle protein D1 and positively regulates the PI3K/AKT-induced epithelial–mesenchymal transition (EMT) pathway to promote the migration and invasion of LUAD cells (50). The remaining four members were KDELR2, MAP3K1, SMC4, and WEE1. The KDEL receptor family includes a transmembrane domain protein called KDEL endoplasmic reticulum protein retention receptor 2 (KDELR2). KDELR2 accelerates the development of breast cancer, non-small cell lung cancer, bladder cancer, and GBM (51–54). According to research, KDELR2 is significantly expressed in GBM tissues and controls mTOR’s degree of phosphorylation (Ser2448), which encourages the growth of GBM tumors (52). Invasion and metastasis of cells are dependent on KDELR2-regulated Golgi secretion, and KDELR2 suppression lessens lung cancer metastasis, according to a new study (51). Serine/threonine kinase MAP3K1 belonging to the MAP3K family is a component of multiple signaling cascades and includes the ERK, JNK, and NF-κB signaling pathways. It is triggered by a multitude of stimuli, including cellular stress, growth hormones, and cytokines (55). Research has shown elevated MAP3K1 expression in GBMs, which is related to poor prognosis and treatment resistance (56). *MAP3K1* promotes cell proliferation and invasion in esophageal cancer, and inhibits anoikis, thus playing a tumor-promoting role (57). The present work, therefore, suggests that LGG patients with a high *MAP3K1* expression demonstrate a poor prognosis which is consistent with earlier reports. An SMC family member referred to as structural maintenance of chromosome 4 (SMC4) encodes the SMC4 protein, which has an elevated expression in a variety of malignancies and may play an oncogenic function (58–62). SMC4 increases the migration, proliferation, and invasion of glioma cells in GBMs *via* acting downstream of MiR-433-3p (60). *WEE1* has an elevated expression in a number of cancer types, including adult GBMs, and breast, colon, and stomach cancers. High *WEE1* expression is linked to poor prognostic indicators (63–66). Interestingly, *SMC4* and *WEE1* were closely associated with the cell cycle (67, 68), and our results from the GSVA analysis of macrosubtypes and GO/KEGG enrichment analysis of DEGs also suggested that the cell cycle is a potentially relevant pathway for anoikis in LGG. This may provide a theoretical basis for further exploration of anoikis in LGGs.

In summary, we have provided a new molecular typing and scoring system for patients with LGG based on anoikis. Our anoikis scoring system illustrates that higher anoiS may cause poorer prognosis, but at the same time brings the possibility of improved responsiveness to TMZ and immunotherapy in LGGs. This paradox may arise from the reduced proportion of IDH mutations and 1p19q co-deletions, because IDH mutations promote immune escape, leading to poor immunotherapy responsiveness, or from the influence of other underlying biological mechanisms of anoikis, such as regulation of the cell cycle. Nonetheless, it is anticipated that this new scoring system would result in more precise prognosis prediction, enhanced clinical diagnosis, and improved therapeutic approaches for individuals with LGG.

This work has a few limitations. Initially, further cohorts of TMZ therapy and immunotherapy are required to confirm the findings and enhance the score system’s prediction ability. Second, the study should take into account the surgical resection margin, a significant clinical determinant of LGGs. Lastly, this study applied lots of correlation analysis to explore the association and closeness between the variables, but could not exclude the existence of nonlinear relationship between the variables and could not determine the causal relationship of the variables.

## 5 Conclusion

To correctly forecast the prognosis of patients with LGG and evaluate their responsiveness to temozolomide and immunotherapy, a new molecular type and scoring system for LGG based on anoikis was developed in this work. Hence, creating more precise, tailored treatment strategies for individuals suffering from LGG seems potential.

## Data availability statement

The datasets presented in this study can be found in online repositories. The names of the repository/repositories and accession number(s) can be found in the article/**Supplementary Material**.

## Author contributions

KC designed the study, GZ and AC conducted the data analysis, and wrote the manuscript. GZ, AC, and JF participated in and contributed to the experiments of this study. JF, AW, GC, PT, HC, and XC participated in manuscript revision. All authors contributed to the article and approved the submitted version.

## Supplementary material

Supplementary Figure 1 Differential expression and prognostic analysis of 19 ANOIRGs in TCGA-LGG cohort. **(A)** Differential expression of 19 ANOIRGs in LGG and normal tissue. **(B)** Prognostic correlation network of 19 ANOIRGs. The line represents the correlation between genes, the sphere represents the univariateCox test of each gene. **(C)** K-M survival analysis of 18 ANOIRGs in LGGs (OS, Log-rank test, p < 0.001). ANOIRGs, anoikis-related genes; * p < 0.05, ** p < 0.01, ***p < 0.001.

Supplementary Figure 2 Genetic variation landscape of 19 ANOIRGs in TCGA-LGG cohort. **(A)** The Waterfall chart showing the types and frequencies of genetic variation in 19 ANOIRGs. **(B)** Frequencies of gain and loss of CNV in 19 ANOIRGs. **(C)** The Chromosome localization and the landscape of CNV in 19 ANOIRGs. ANOIRGs, anoikis-related genes.

Supplementary Figure 3 Detailed results of consensus clustering. **(A–C)** Detailed results of consensus clustering to identifity anoikis related subtypes based on ANOIRGs: cumulative distribution curve **(A)**, area under the cumulative distribution curve **(B)** and tracking plot **(C)**. **(D–F)** Detailed Results of consensus clustering to construct gene-subtypes based on SDEPGs: cumulative distribution curve **(D)**, area under the cumulative distribution curve **(E)** and tracking plot **(F)**. ANOIRGs, anoikis-related genes; SDEPGs, differentially expressed prognostic genes with strong prognostic significance.

Supplementary Figure 4 Difference comparison of enriched pathways of four different anoikis related subtypes by GSVA. **(A–C)** Heatmaps comparing GSVA pathway scores for four anoikis related subtypes from three items: KEGG **(A),** HALLMARK **(B)**, and Reactome **(C)**.

Supplementary Figure 5 Tumor immune microenvironment analysis of four anoikis related subtypes. **(A)** t-SNE analysis of the four subtypes based on ANOIRGs expression **(B)** Differential analysis of TME scores between the four anoikis related subtypes. **(C)** The infiltration abundance of 23 kinds of immune cells was evaluated by ssGSEA in the four anoikis related subtypes. ANOIRGs, anoikis-related gene; *p < 0.05; **p < 0.01; and ***p < 0.001.

Supplementary Figure 6 Construction of anoiS and grouping based on anoiS. **(A)** Screening of optimal parameter (lambda) and ANOIRGs with non-zero coefficients. When the number of genes with non-zero coefficients was 34, the Lasso model was the most stable. **(B)** Lasso coefficient profiles of the 669 SPDEGs. **(C)** The relationship between the number of trees and model error in random forest. The model has the smallest error when the number of trees is 303. **(D)** The top 30 genes were ranked by gene importance score based on Gini coefficient method. **(E)** Sankey diagram to show the correspondence among anoikis related subtypes, gene-subtypes, anoiS and survival outcomes. **(F)** Differential analysis in anoiS levels between the four anoikis related subtypes. **(G)** Differential analysis in anoiS levels between the two gene-subtypes. anoiS, anoikis riskscore; ANOIRGs, anoikis-related genes; SPDEGs, differentially expressed prognostic genes with strong prognostic significance.

Supplementary Figure 7 Gene expression differential analysis and survival analysis of 12 APRGs in LGGs. **(A)** Gene expression differential analysis of 12 APRGs between LGG and normal tissue. **(B)** K-M survival analysis of 12 APRGs in LGGs (OS, Log-rank test, p < 0.001). APRGs, anoikis-potential related genes.

Supplementary Figure 8 Spearman Correlation analysis among TME, anoiS and 12 APRGs. **(A)** Correlation scatter plot between anoiS and infiltration level of 21 significative immune cells. **(B)** Correlation matrix between expression level of 12 APRGs and infiltration level of 23 immune cells. anoiS, anoikis riskscore; APRGs, anoikis-potential related genes; *p < 0.05; **p < 0.01; and ***p < 0.001.

## Glossary

**Table:**

AKGs	anoikis, key, genes
ANOIRGs	anoikis-related, genes
anoiS	anoikis, riskscore
APRGs	anoikis, potentially, relevant, genes
AUC	area, under, the, curve
*CCT5*	chaperonin, containing, TCP1, subunit, 5
CGGA	China, Glioma, Genome, Atlas
CNVs	copy, number, variants
COX-2	cyclooxygenase-2
DEGs	differentially, expressed, genes
ECM	cell-extracellular, matrix
FPKM	fragments, per, kilobase, million
GBMs	glioblastomas
GSEA	gene, set, enrichment, analysis
GSVA	Gene, Set, Variation, Analysis
GTEx	Genotype-Tissue, Expression, Project
HPA	Human, Protein, Atlas
ICGs	immune, checkpoint, genes
IDH	isocitrate, dehydrogenase
IHC	immunohistochemical
*KDELR2*	KDEL, endoplasmic, reticulum, protein, retention, receptor, 2
KEGG	Kyoto, Encyclopedia, of, Genes, and, Genomes
K-M	Kaplan–Meier
LASSO	Least, Absolute, Shrinkage, and, Selection, Operator
LGGs	low-grade, gliomas
MAF	mutation, annotation, format
MDSCs	myeloid-derived, suppressor, cells
MGMT	O^6^-methylguanine-DNA, methyltransferase
OS	overall, survival
PFS	progression-free, survival
qRT-PCR	quantitative, reverse, transcription, polymerase, chain, reaction
ROC	receiver, operating, characteristic
SDEPGs	differentially, expressed, prognostic, genes, with, strong, prognostic, significance
*SMC4*	Structural, maintenance, of, chromosome, 4
ssGSEA	single-sample, gene, set, enrichment, analysis
TCGA	The, Cancer, Genome, Atlas
TIME	The, tumor, immune, microenvironment
TMB	tumor, mutation, burden
TMZ	temozolomide
TPM	transcripts, per, kilobase, million
t-SNE	t-Distributed, Stochastic, Neighbor, Embedding
