# Supplementary figures and images for: Construction of a novel molecular typing and scoring system for anoikis distinguishes between different prognostic risks and treatment responsiveness in low-grade glioma
Source: Front Immunol. 2023 Apr 11;14:1105210. doi: 10.3389/fimmu.2023.1105210 (PMC10126347; doi:10.3389/fimmu.2023.1105210)

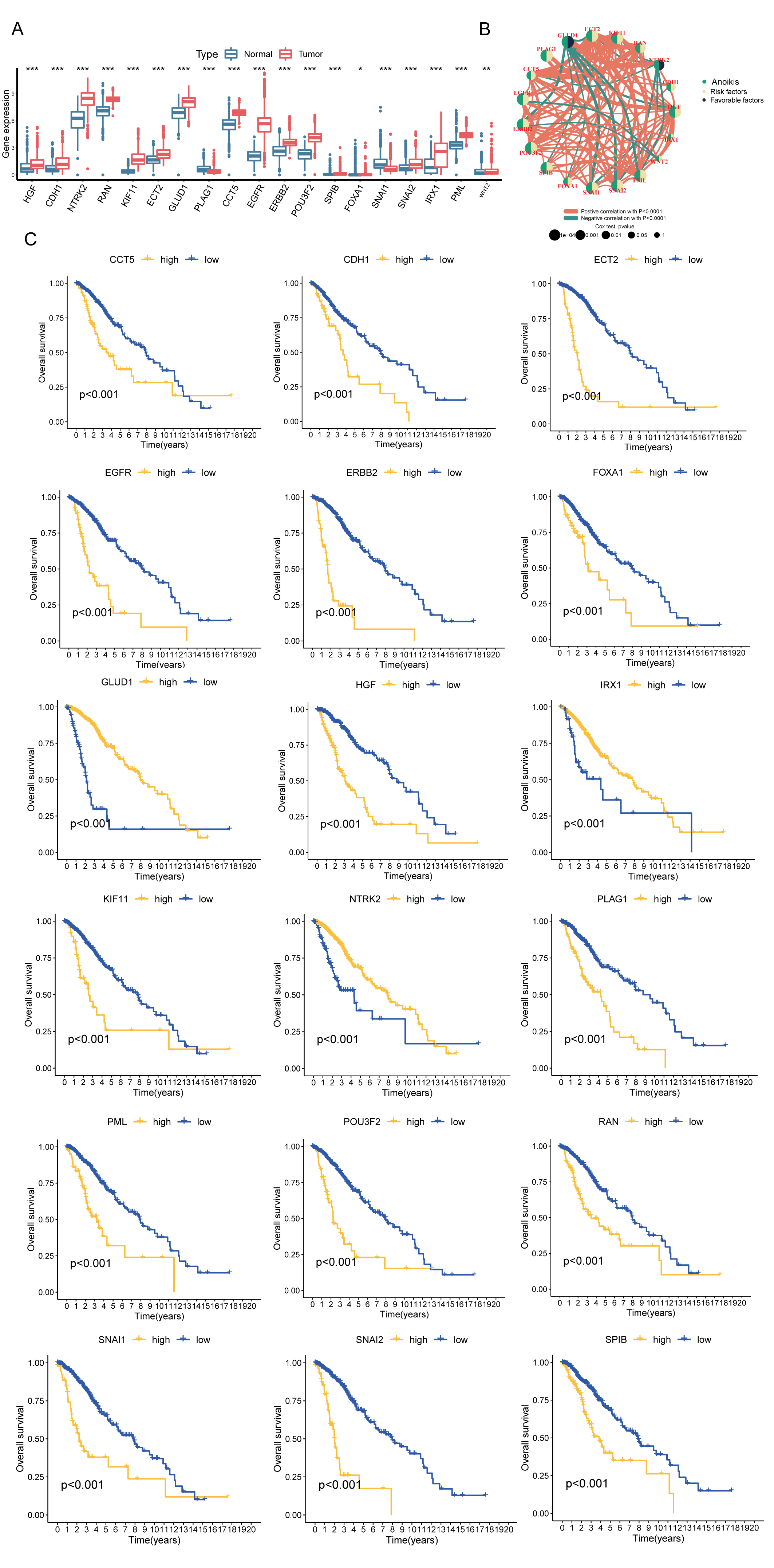

Supplement: Supplementary Figure 1 — Differential expression and prognostic analysis of 19 ANOIRGs in TCGA-LGG cohort. (A) Differential expression of 19 ANOIRGs in LGG and normal tissue. (B) Prognostic correlation network of 19 ANOIRGs. The line represents the correlation between genes, the sphere represents the univariateCox test of each gene. (C) K-M survival analysis of 18 ANOIRGs in LGGs (OS, Log-rank test, p < 0.001). ANOIRGs, anoikis-related genes; * p < 0.05, ** p < 0.01, ***p < 0.001. [file DataSheet_1.zip › Supplementary Figures/Supplementary Figure-1.tif]

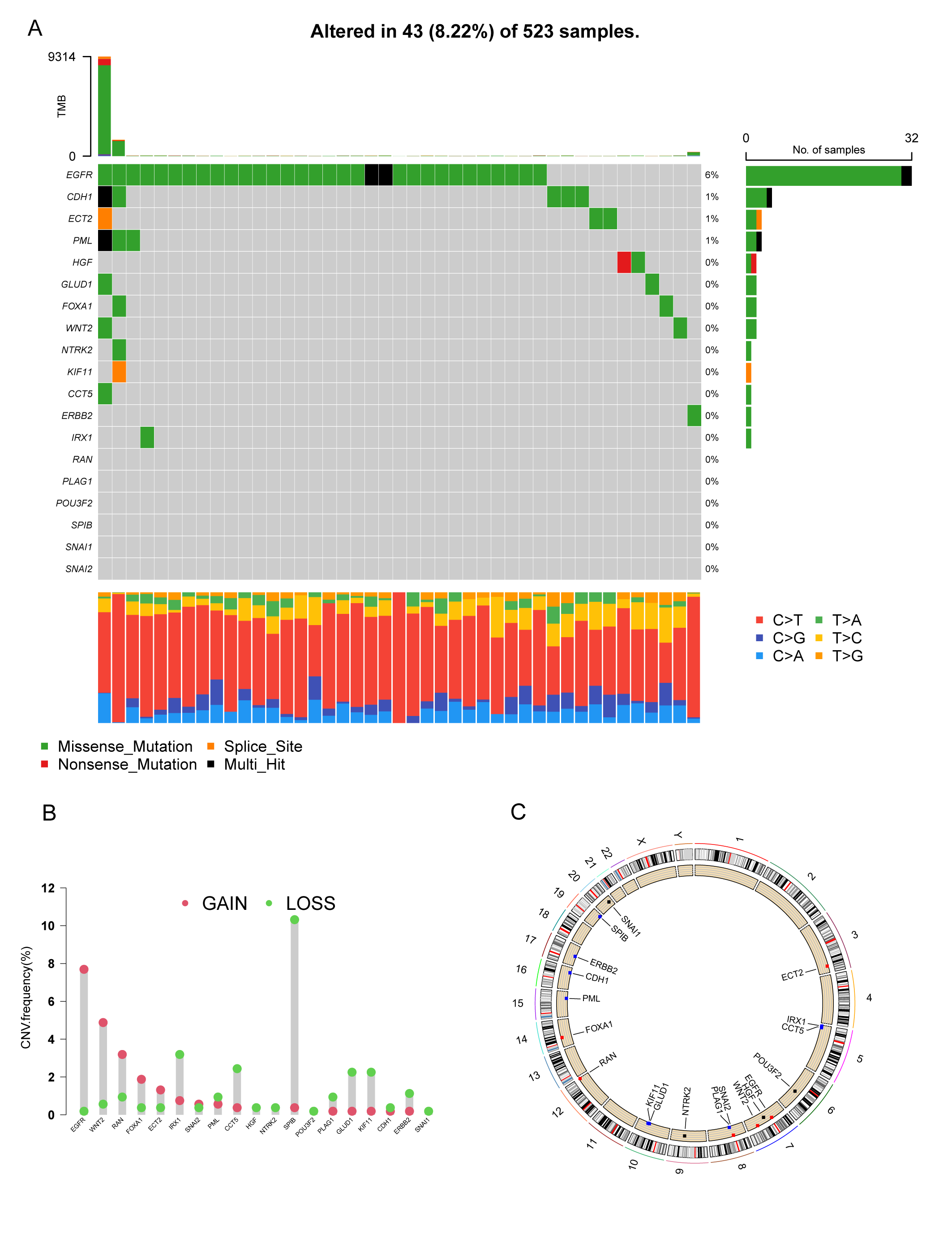

Supplement: Supplementary Figure 1 — Differential expression and prognostic analysis of 19 ANOIRGs in TCGA-LGG cohort. (A) Differential expression of 19 ANOIRGs in LGG and normal tissue. (B) Prognostic correlation network of 19 ANOIRGs. The line represents the correlation between genes, the sphere represents the univariateCox test of each gene. (C) K-M survival analysis of 18 ANOIRGs in LGGs (OS, Log-rank test, p < 0.001). ANOIRGs, anoikis-related genes; * p < 0.05, ** p < 0.01, ***p < 0.001. [file DataSheet_1.zip › Supplementary Figures/Supplementary Figure-2.tif]

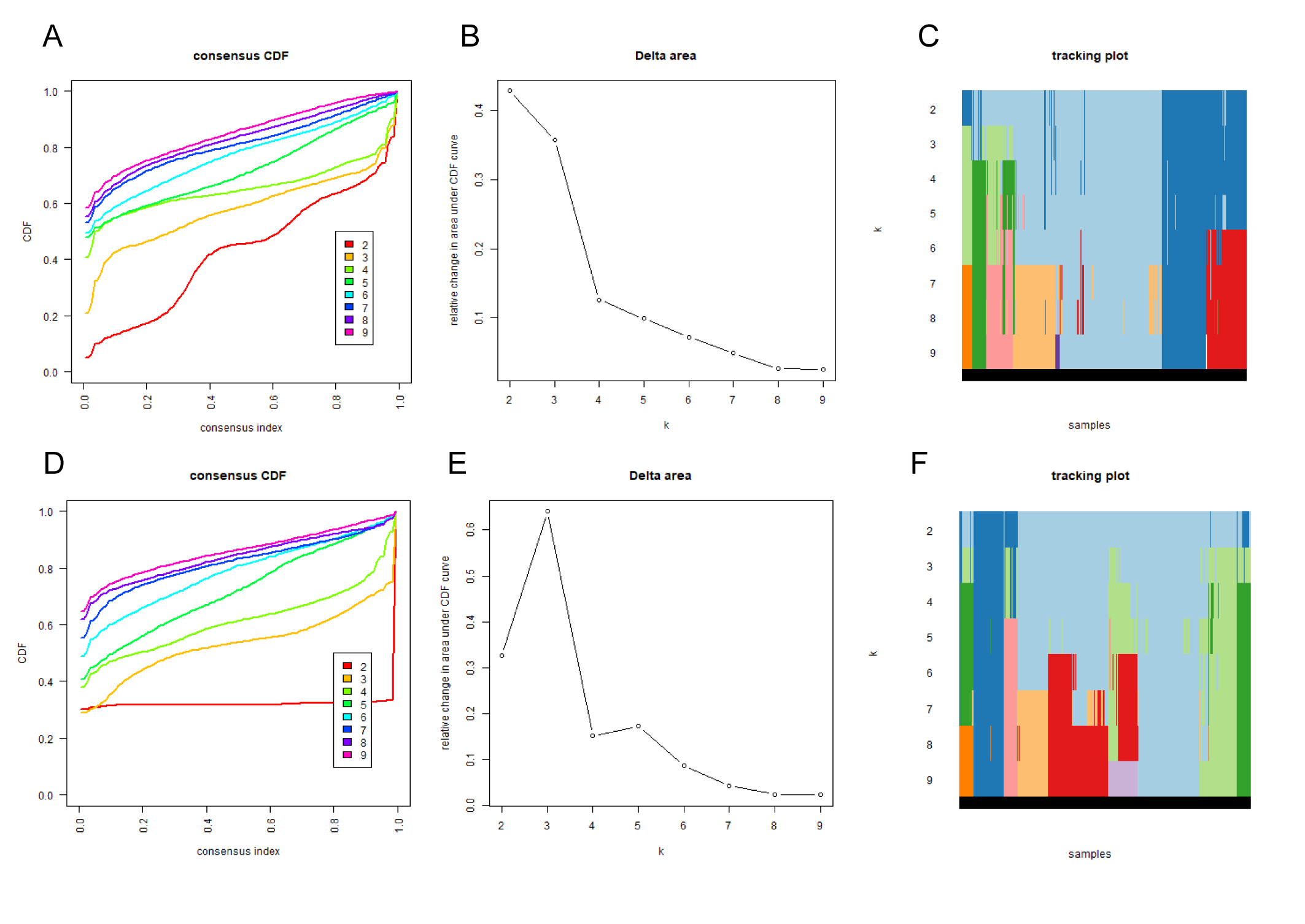

Supplement: Supplementary Figure 1 — Differential expression and prognostic analysis of 19 ANOIRGs in TCGA-LGG cohort. (A) Differential expression of 19 ANOIRGs in LGG and normal tissue. (B) Prognostic correlation network of 19 ANOIRGs. The line represents the correlation between genes, the sphere represents the univariateCox test of each gene. (C) K-M survival analysis of 18 ANOIRGs in LGGs (OS, Log-rank test, p < 0.001). ANOIRGs, anoikis-related genes; * p < 0.05, ** p < 0.01, ***p < 0.001. [file DataSheet_1.zip › Supplementary Figures/Supplementary Figure-3.tif]

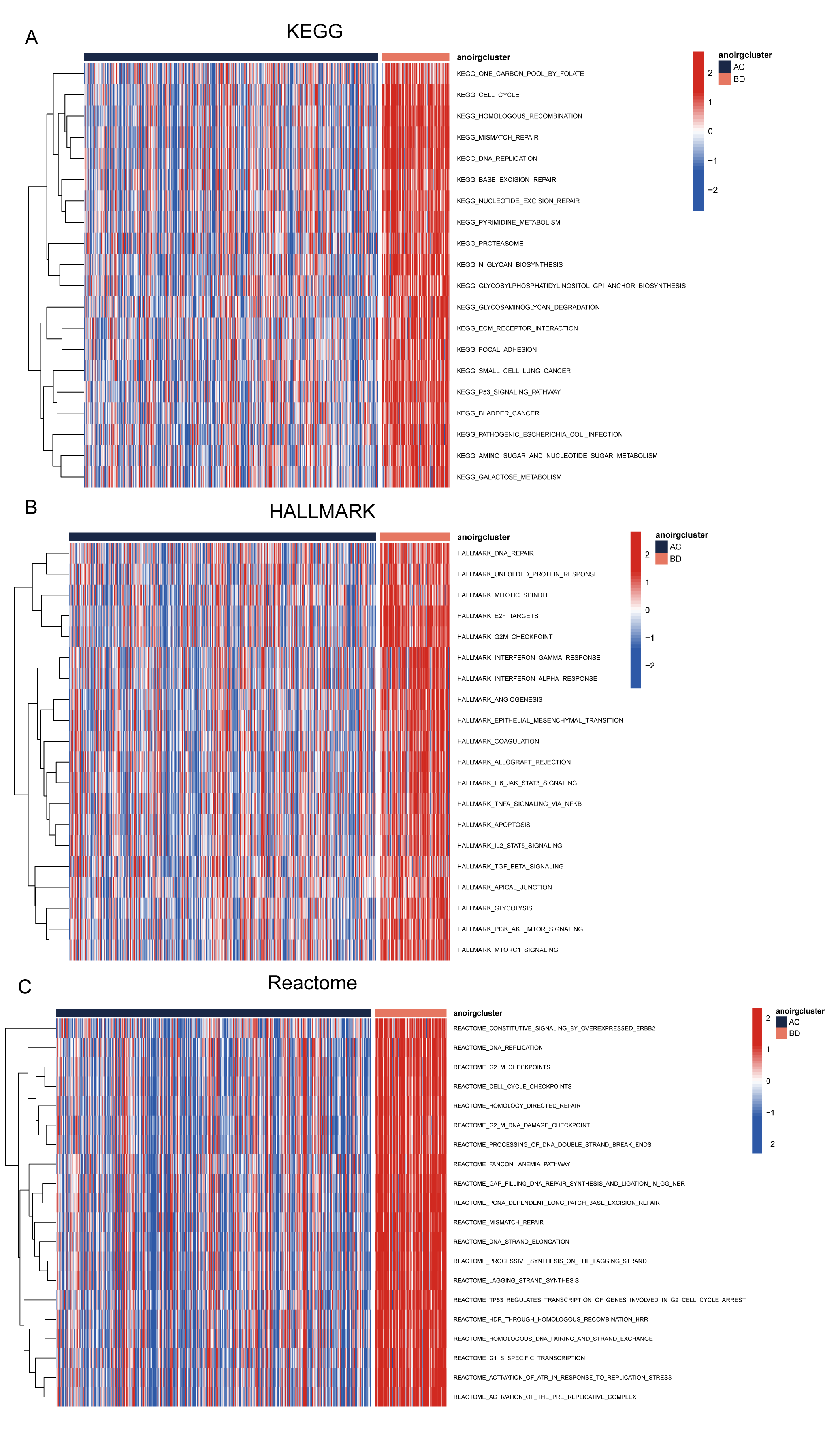

Supplement: Supplementary Figure 1 — Differential expression and prognostic analysis of 19 ANOIRGs in TCGA-LGG cohort. (A) Differential expression of 19 ANOIRGs in LGG and normal tissue. (B) Prognostic correlation network of 19 ANOIRGs. The line represents the correlation between genes, the sphere represents the univariateCox test of each gene. (C) K-M survival analysis of 18 ANOIRGs in LGGs (OS, Log-rank test, p < 0.001). ANOIRGs, anoikis-related genes; * p < 0.05, ** p < 0.01, ***p < 0.001. [file DataSheet_1.zip › Supplementary Figures/Supplementary Figure-4.tif]

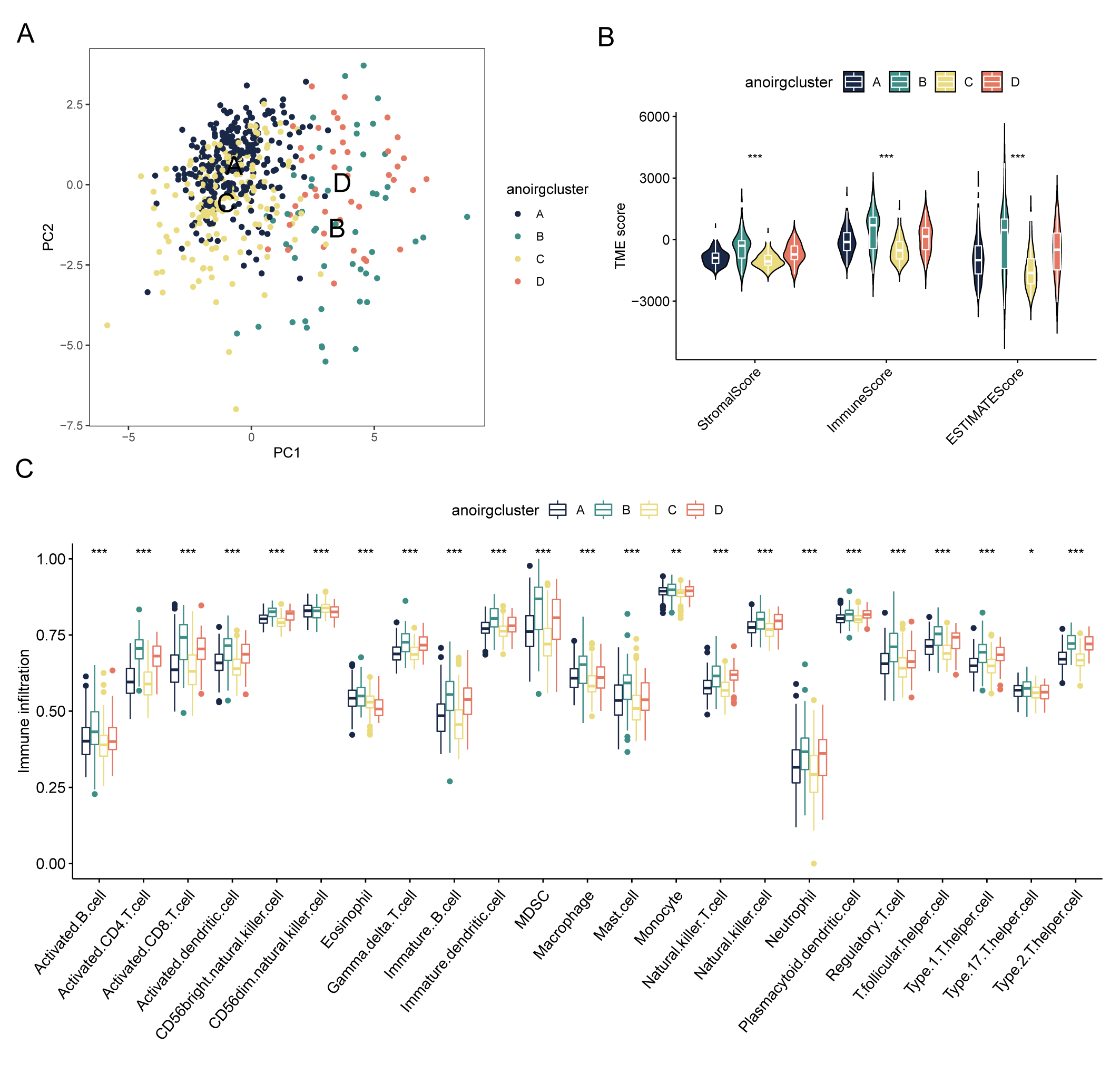

Supplement: Supplementary Figure 1 — Differential expression and prognostic analysis of 19 ANOIRGs in TCGA-LGG cohort. (A) Differential expression of 19 ANOIRGs in LGG and normal tissue. (B) Prognostic correlation network of 19 ANOIRGs. The line represents the correlation between genes, the sphere represents the univariateCox test of each gene. (C) K-M survival analysis of 18 ANOIRGs in LGGs (OS, Log-rank test, p < 0.001). ANOIRGs, anoikis-related genes; * p < 0.05, ** p < 0.01, ***p < 0.001. [file DataSheet_1.zip › Supplementary Figures/Supplementary Figure-5.tif]

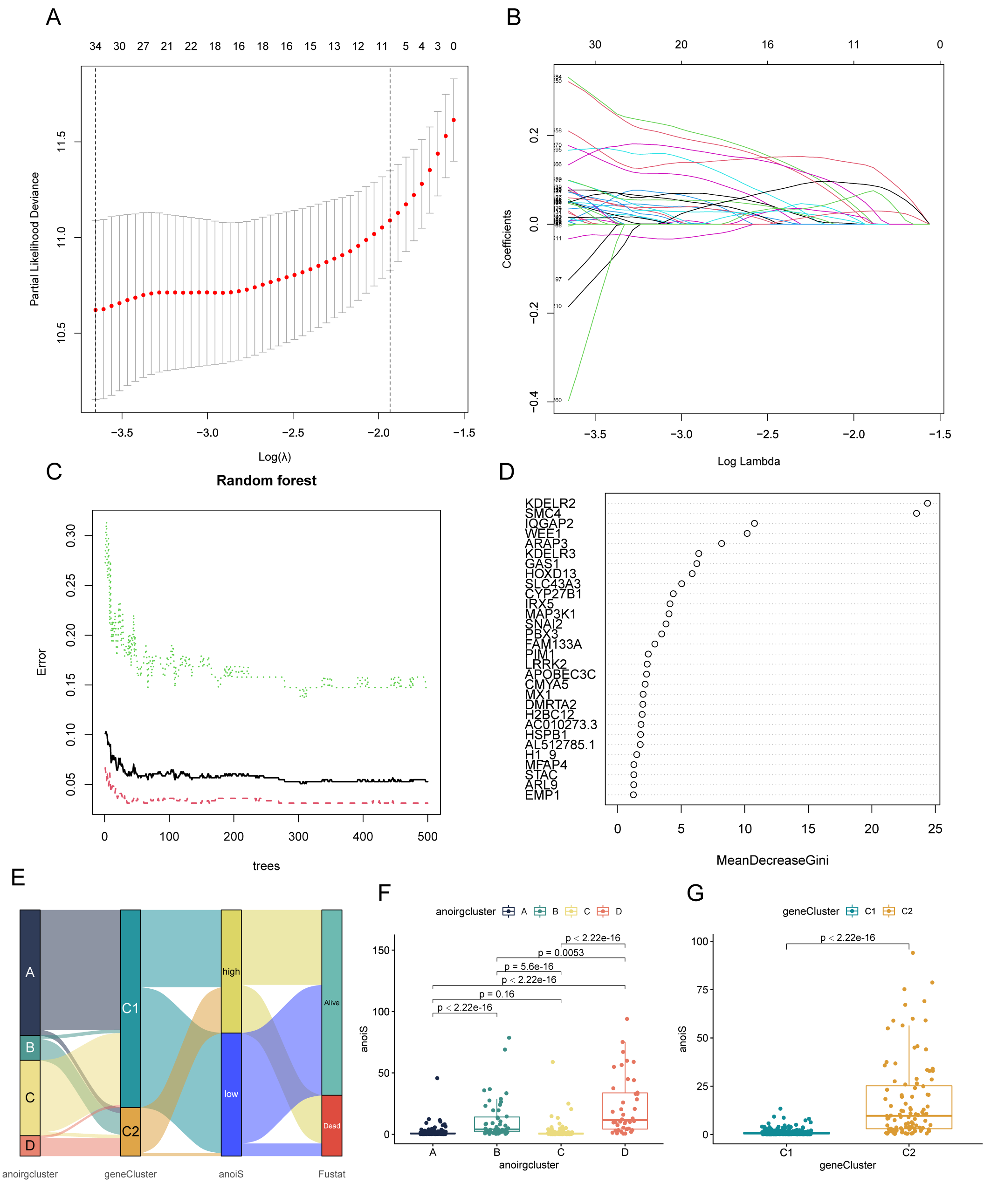

Supplement: Supplementary Figure 1 — Differential expression and prognostic analysis of 19 ANOIRGs in TCGA-LGG cohort. (A) Differential expression of 19 ANOIRGs in LGG and normal tissue. (B) Prognostic correlation network of 19 ANOIRGs. The line represents the correlation between genes, the sphere represents the univariateCox test of each gene. (C) K-M survival analysis of 18 ANOIRGs in LGGs (OS, Log-rank test, p < 0.001). ANOIRGs, anoikis-related genes; * p < 0.05, ** p < 0.01, ***p < 0.001. [file DataSheet_1.zip › Supplementary Figures/Supplementary Figure-6.tif]

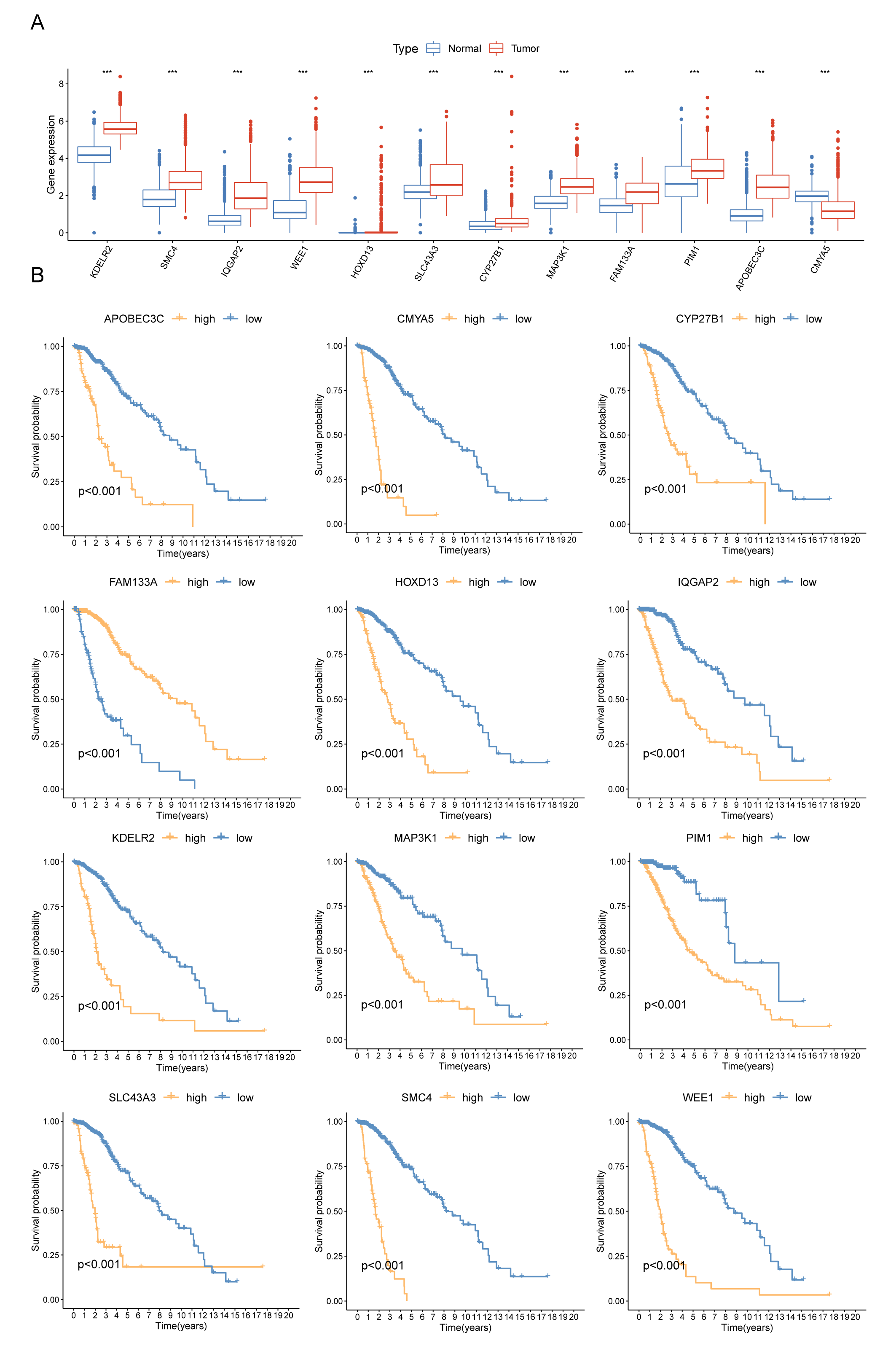

Supplement: Supplementary Figure 1 — Differential expression and prognostic analysis of 19 ANOIRGs in TCGA-LGG cohort. (A) Differential expression of 19 ANOIRGs in LGG and normal tissue. (B) Prognostic correlation network of 19 ANOIRGs. The line represents the correlation between genes, the sphere represents the univariateCox test of each gene. (C) K-M survival analysis of 18 ANOIRGs in LGGs (OS, Log-rank test, p < 0.001). ANOIRGs, anoikis-related genes; * p < 0.05, ** p < 0.01, ***p < 0.001. [file DataSheet_1.zip › Supplementary Figures/Supplementary Figure-7.tif]

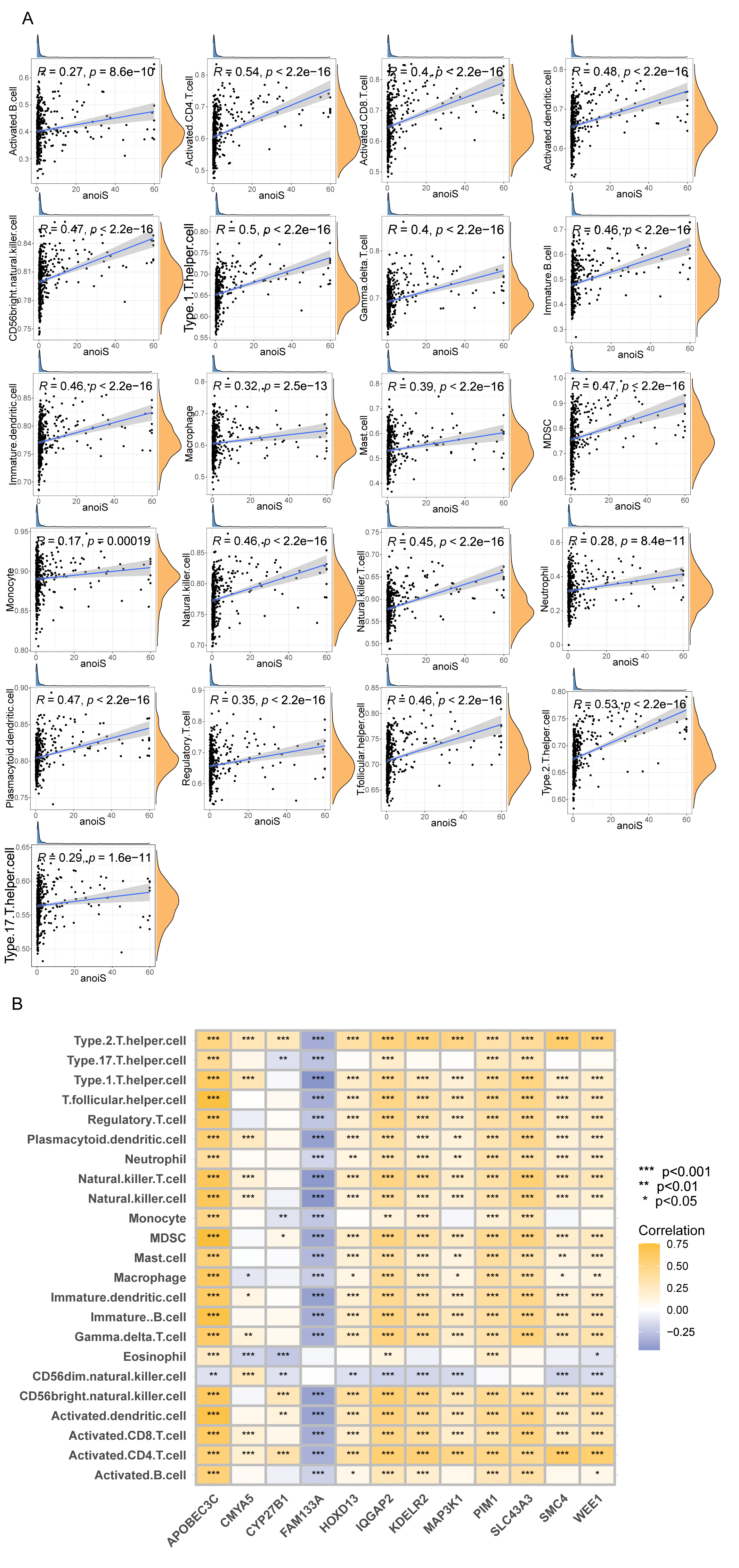

Supplement: Supplementary Figure 1 — Differential expression and prognostic analysis of 19 ANOIRGs in TCGA-LGG cohort. (A) Differential expression of 19 ANOIRGs in LGG and normal tissue. (B) Prognostic correlation network of 19 ANOIRGs. The line represents the correlation between genes, the sphere represents the univariateCox test of each gene. (C) K-M survival analysis of 18 ANOIRGs in LGGs (OS, Log-rank test, p < 0.001). ANOIRGs, anoikis-related genes; * p < 0.05, ** p < 0.01, ***p < 0.001. [file DataSheet_1.zip › Supplementary Figures/Supplementary Figure-8.tif]
