# Supplementary material for: Construction of a novel molecular typing and scoring system for anoikis distinguishes between different prognostic risks and treatment responsiveness in low-grade glioma
Source: Front Immunol. 2023 Apr 11;14:1105210. doi: 10.3389/fimmu.2023.1105210 (PMC10126347; doi:10.3389/fimmu.2023.1105210)
Supplement: Supplementary file 2 [file Table_1.docx]

**Supplementary Table 1**| Univariate Cox regression analysis of the 12 APRGs.

| Gene | HR (95%CI low-95%CI high) | p value |
| --- | --- | --- |
| KDELR2 | 2.47 (1.93-3.17) | 9.70E-13 |
| SMC4 | 2.26 (1.93-2.64) | 1.39E-24 |
| IQGAP2 | 1.73 (1.54-1.95) | 5.07E-20 |
| WEE1 | 2.18 (1.88-2.53) | 2.73E-25 |
| HOXD13 | 1.72 (1.54-1.92) | 1.17E-21 |
| SLC43A3 | 1.57 (1.38-1.79) | 6.98E-12 |
| CYP27B1 | 1.50 (1.36-1.66) | 4.71E-16 |
| MAP3K1 | 2.14 (1.77-2.58) | 2.89E-15 |
| FAM133A | 0.48 (0.41-0.56) | 2.07E-21 |
| PIM1 | 1.80 (1.51-2.15) | 4.07E-11 |
| APOBEC3C | 2.08 (1.77-2.46) | 4.06E-18 |
| CMYA5 | 2.12 (1.85-2.42) | 2.73E-28 |
