# Supplementary material for: Construction of a novel molecular typing and scoring system for anoikis distinguishes between different prognostic risks and treatment responsiveness in low-grade glioma
Source: Front Immunol. 2023 Apr 11;14:1105210. doi: 10.3389/fimmu.2023.1105210 (PMC10126347; doi:10.3389/fimmu.2023.1105210)
Supplement: Supplementary file 3 [file Table_2.docx]

**Supplementary Table 2** **|** Information of primer sequence in qRT-PCR.

| Gene Symbol | NCBI ID | Sequence（5' to 3'） | |
| --- | --- | --- | --- |
| CCT5 | 22948 | F | TGCTGGTCATCGAGCAGTGT |
|  |  | R | GGGCACTTATCCGCCTCTTG |
| MAP3K1 | 4214 | F | TGGAGTGTTGGCTGTGCTATT |
|  |  | R | AGCCACATCTCGTAAACCAGG |
| SMC4 | 10051 | F | CTTACTTTGGGAGGGGACGC |
|  |  | R | GTAAAGGGGAGTGGGCTTGT |
| KDELR2 | 11014 | F | TGGGAAAAGCCAGCTTCTGT |
|  |  | R | CCACCAGAAACTCCACTCGG |
| WEE1 | 7465 | F | GCTTGCCCTCACAGTGGTAT |
|  |  | R | GCACTTGTGGTATCCGAGGT |

Notes: F: Forward; R: Reverse.
